# Sequential regulation of dual catalytic modules for the directed biosynthesis of 2,5-furandicarboxylic acid in *Escherichia coli*

**DOI:** 10.1186/s40643-026-01159-5

**Published:** 2026-11-07

**Authors:** Fan Yang, Yan Li, Xinrui Qi, Huanan Li, Wanli Cheng, Jiashu Liu, Zhengbing Jiang

**Affiliations:** 1 School of Life Science, Hubei University, Wuhan, 430062 People’s Republic Of China; 2 Hubei Key Laboratory of Industrial Biotechnology, Hubei University, Wuhan, 430062 People’s Republic Of China

**Keywords:** Biocatalysis, 2,5-Furandicarboxylic acid, 5-Hydroxymethylfurfural, Sequential regulation of dual catalytic modules, Life cycle assessment

## Abstract

Bioconversion of 5-hydroxymethylfurfural (HMF) to 2,5-furandicarboxylic acid (FDCA) confers advantages of mild conditions, high selectivity, and eco-friendliness. However, enzymatic catalysis is expensive and multi-strain microbial catalysis is limited by the transmembrane transport of intermediates between strains, restricting the efficient synthesis of FDCA and generating a byproduct. In this study, we aimed to establish sequential regulation of dual catalytic modules in *Escherichia coli* to precisely control the expression of hydroxymethylfurfural oxidase (HMFO) and vanillin dehydrogenase (VDH1), enabling the directed one-pot conversion of HMF to FDCA with no detectable accumulation of the measured intermediates and simplifying the operational process. At a 15 mM HMF scale, this strategy achieved a FDCA yield of 100%, significantly higher than the 78.6% yield of the dual-enzyme cascade system established by HMFO and VDH1, and the 56.73% yield from the dual-strain catalysis constructed by two engineered *E. coli* strains that harbor either *hmfo* or *vdh*1(Student’s *t*-test, *p* < 0.0001). Furthermore, at a laboratory scale (production of a fermentation broth containing 100 g of FDCA), life cycle assessment demonstrated sequential regulation of dual catalytic modules represented the lowest environmental impact among the three systems (dual-strain catalysis and dual-enzyme cascade). It reduced global warming potential, fossil fuel depletion, and ecotoxicity by 70–82% compared with the other two systems. Overall, this study establishes a more efficient and sustainable strategy for directed FDCA biosynthesis.

**Supplementary Information:**

The online version contains supplementary material available at https://doi.org/10.1186/s40643-026-01159-5.

## Introduction

In recent years, bio-based materials have gained increasing interest owing to their renewability and environmental friendliness. In 2004, the US Department of Energy selected 12 representative bio-based platform chemicals, among which 2,5-furandicarboxylic acid (FDCA) was the primary aromatic representative (Werpy 2004). FDCA serves as a key precursor for bio-based polyethylene 2,5-furandicarboxylate (PEF), which is a biodegradable alternative to the widely used, high-yielding polyethylene terephthalate (PET) (Cajnko et al. 2020). Compared with PET, PEF confers superior biodegradability, renewability, recyclability, gas barrier and mechanical properties, tensile strength, and thermal performance (Stegmann et al. 2023). FDCA production is generally initiated by the oxidation of 5-hydroxymethylfurfural (HMF) (Chen et al. 2018, Han et al. 2016). Several catalytic methods for converting HMF to FDCA have been reported, including chemical catalysis, electrochemical catalysis (Kubota and Choi 2018; Sajid et al. 2018), and biocatalysis. Compared with chemical and electrochemical catalysis, biocatalysis achieves high selectivity in converting HMF in mild aqueous solution, avoiding the use of noble metal catalysts, organic solvents, and high temperatures and pressures. Consequently, FDCA biosynthesis presents a promising strategy to substantially reduce energy consumption and carbon emissions (Yang et al. 2023, Chang et al. 2023).

Currently, FDCA biosynthesis is achieved using three strategies: enzymatic catalysis, microbial catalysis, and combined microbial and enzymatic catalysis (Yang et al. 2023, Sayed et al. 2025, Karich et al. 2018). The complete oxidation of HMF to FDCA involves the stepwise oxidation of its alcohol and aldehyde functional groups, and significant progress has been made in each of these approaches (Table S1). In enzymatic catalysis, complete HMF oxidation often necessitates a combination of two or three enzymes or mediators, combinations such as laccase with (2,2,6,6-tetramethyl-piperidin-1-yl)oxyl (Wang et al. 2018), aryl-alcohol oxidase (AAO) with an unspecific peroxygenase (Carro et al. 2015), and galactose oxidase (GOase) with lipase have been reported (Qin et al. 2015); combinations such as laccase with alcohol oxidase (Yang et al. 2023) and lipase with alcohol oxidase (Li et al. 2025) were reported in our previous study. However, the addition of mediators and purified enzymes in these approaches increases overall cost and environmental impact (Yang et al. 2025). Microorganism–enzyme combinations have also been used in FDCA synthesis, such as *Trichoderma reesei* with laccase (Troiano et al. 2022) and *Pseudomonas putida* KT2440 with laccase (Zou et al. 2020). Nevertheless, this strategy poses challenges owing to its complex operation and high cost. Microbial catalysis does not require mediators and enzyme purification. Moreover, enzymes exhibit superior stability and reduced sensitivity to toxic substrates within the cell (McKenna et al. 2015), making microbial catalysis a promising strategy for FDCA biosynthesis. Microbial catalysis can be performed using either a single strain or multi-strain catalysis. In multi-strain catalysis, the pathway is divided among different strains. For instance, *E. coli* M overexpressing the *GOase* gene, in combination with *Pseudomonas putida* KT2440 (Tan et al. 2020), catalyzes the conversion of HMF to FDCA. However, this process is associated with high cost, operational complexity, and inefficient transmembrane transport of intermediates, which limits efficient FDCA synthesis. In single-strain biosynthesis, the requisite enzymes are expressed within a single cell, where the substrate can be efficiently converted to the final product. A previous study reported engineered *Escherichia coli* overexpressing the AAO gene could convert 2,5-dicarboxaldehydefuran (DFF) to FDCA (Yang et al. 2025). Although the single-strain biosynthesis of FDCA is characterized by low cost and simple operation, the pathway from HMF to FDCA cannot be precisely directed, which often leads to the accumulation of intermediates. Therefore, a novel FDCA synthesis strategy was constructed to precisely control the pathway, thereby achieving both high catalytic efficiency and complete conversion of HMF to FDCA.

Sequential regulation of dual catalytic modules enables real-time, precise control of gene expression timing and intensity in a single strain. The T7 expression system is widely used owing to its high expression efficiency and strong specificity; transcription is activated by the addition of isopropyl β-D-1-thiogalactopyranoside (IPTG) (Lin et al. 2024). When culture temperature exceeds 37 °C, the binding affinity of T7 RNA polymerase to the P_*T7*_ promoter weakens (Boulain et al. 2013), resulting in decreased expression. Therefore, sequential regulation of dual catalytic modules based on the λpL/pR-cI857 thermally inducible system and the T7 expression system can be established in a single strain. By simply adjusting culture temperature, this strategy can be applied to achieve coordinated control over the expression of two or more genes involved in converting HMF to FDCA.

As global concern on environmental sustainability increases, it is crucial to simultaneously consider FDCA productivity and environmental implications when designing a biosynthesis strategy. Life cycle assessment (LCA) is a tool to assess the potential environmental impacts and resources used throughout a product’s life cycle (Bjørn et al. 2018). Previous studies have reported that different microbial strains, chemical dosage, and electrical input power during biosynthetic processes can lead to markedly different environmental impacts (Qin et al. 2023, Hobusch et al. 2024, Feijoo et al. 2017). Given these differences, it is necessary to evaluate the environmental impacts of FDCA production strategies from a comprehensive perspective to promote globally sustainable development. Therefore, we used LCA to compare current strategies for FDCA biosynthesis.

In this study, we aimed to achieve the directed biosynthesis of FDCA in *E. coli* through sequential regulation of dual catalytic modules, and it was realized based on the λpL/pR-cI857 thermally inducible system and the T7 expression system. Its effectiveness was verified by incorporating the gene encoding green fluorescent protein (GFP; *gfp*) and mCherry red fluorescent protein (mCherry; *mcherry*). To convert HMF into FDCA, we replaced *gfp* and *mcherry* with the gene encoding hydroxymethylfurfural oxidase (HMFO; *hmfo*) (Dijkman and Fraaije 2014) from *Methylovorus* sp. strain MP 688 and the gene encoding vanillin dehydrogenase (VDH1; *vdh*1) (Zhu et al. 2023) from *Comamonas testosteroni* strain SC1588. Subsequently, we analyzed the relative transcription levels of *hmfo* and *vdh*1 in dual catalytic modules. We optimized the conditions for converting HMF to FDCA within sequential regulation of dual catalytic modules. Moreover, we analyzed and compared the FDCA production among the three strategies, including the dual-enzyme cascade system (utilizing HMFO and VDH1), the dual-strain catalysis system (incorporating the *hmfo* and *vdh*1 genes), and sequential regulation of dual catalytic modules. The production process was comprehensively evaluated using LCA.

## Experimental section

### Strains, plasmids, and materials

*E. coli* DH5α competent cells were used for cloning plasmids. Plasmid-based switching experiments were performed in *E. coli* BL21 (DE3). The plasmid and strains used in this study are listed in Table S2. HMF, DFF, HMFCA, FFCA, and FDCA were purchased from Shanghai Aladdin Biochemical Technology Co., Ltd. (China).

### Plasmid construction

The plasmid pDawn-*mcherry* served as the template for *mcherry* gene amplification using primers PET-mCherry-F and PET-mCherry-R; *mcherry* was cloned into linearized pET28a (digested with *Bam*H I, *Not* I) by homologous recombination. The plasmid pET28a-*gfp* was used as the template for *gfp* gene amplification using primers PBV-GFP-F, PBV-GFP-R, and the *gfp* gene was cloned into the linearized pBV220 vector (digested with *Bam*H I, *Eco*R I) to construct the pBV-*gfp*. Subsequently, using pBV-*gfp* as the template, the cI857-gfp-rrnB T1 terminator fragment was amplified with primers T7-CI-F and PET-TER-R and cloned into pET28a-*mcherry* that had been linearized by PCR with primers TER-PET-F and CI-T7-R to construct pET28a-*mcherry-gfp*. The primers used in this study are listed in Table S3; the plasmid construction process is shown in Fig. S1.

The genes encoding *vdh*1 from *C. testosteron*i strain SC1588 (GenBank: MN460795) (Zhu et al. 2023) and *hmfo* from *Methylovorus* sp. strain MP688 (GenBank: WP_013440946) (Dijkman and Fraaije 2014) were synthesized according to sequences from the NCBI database; codon optimization for BL21(DE3) was performed by Shanghai Sangon Biotech Co., Ltd. (China). They were subsequently constructed into the pET28a and pET28a-sumo vectors, respectively. *vdh*1 was amplified using primers PBV-VDH-F and PBV-VDH-R and cloned into linearized pBV220 (digestion with *Eco*R I, *Bam*H I) to construct the plasmid pBV220-*vdh*1. Similarly, the cI857-vdh1-rrnB T1 terminator was amplified using primers T7-CI-F and PET-TER-R and cloned into the linearized pET28a-sumo-*hmfo* to construct the plasmid pET28a-sumo-*hmfo-vdh*1. The primers used in this study are listed in Table S3.

### Regulatory switch verification

The plasmid pET28a-*mcherry-gfp* was transformed into BL21 (DE3) to verify the regulatory switch. BL21/pET28a-*mcherry-gfp* was cultured overnight at 30 °C in a lysogeny broth (LB) medium supplemented with kanamycin (50 µg/mL). A seed culture (2%, v/v) was inoculated in LB medium and cultured until OD_600_ reached ≈ 0.6–0.8. GFP induction was performed at 42 °C for 12 h; subsequently, 0.1 mM IPTG was added to induce mCherry at 28 °C for 12 h. Samples were taken every 6 h to detect the fluorescence intensities of mCherry and GFP.

mCherry and GFP fluorescence intensities were detected using the Fluorospectrometer LS-55 (PE, USA). mCherry was detected with an excitation/emission maxima at 587/610 nm (Yin et al. 2013), whereas GFP was detected with an excitation/emission maxima at 488/510 nm (Gorbachev et al. 2020). mCherry and GFP samples were diluted 10-fold and 40-fold, respectively. All experiments were performed in triplicate.

### Real-time quantitative polymerase chain reaction (qPCR) analysis

The plasmid pET28a-sumo-*hmfo-vdh*1 was transformed into BL21 (DE3). Positive transformants were selected from LB plates supplemented with 50 µg/mL kanamycin. BL21/pET28a-sumo-*hmfo-vdh*1 was pre-cultured overnight at 30 °C in a terrific broth (TB) medium supplemented with kanamycin (50 µg/mL). A seed culture (2%, v/v) was then inoculated in a TB medium (containing 50 µg/mL kanamycin) and cultured until OD_600_ reached ≈ 0.6–0.8. VDH1 was induced by alternating incubation between 42 °C for 0.5 h and 37 °C for 3 h, for a total duration of 12 h. Later, HMFO was induced by adding 0.4 mM IPTG at 18 °C for 12 h. BL21/pET28a-sumo-*hmfo-vdh*1 was cultured at 30 °C as the control group; samples were taken to analyze the relative transcription levels of *hmfo* and *vdh*1 at 0, 6, 12, 18 and 24 h.

Following the protocol from our previous study (Li et al. 2024), total RNA was extracted from the samples and reverse-transcribed into cDNA, which was used as the template for qPCR, and 16 S rRNA served as the housekeeping gene for normalization. The primers of the 16 S rRNA reference gene and target gene are listed in Table S3. The relative transcription levels of *hmfo* and *vdh*1 were calculated using the following formula (1):

1 $$\:{2}^{-{\Delta\:}{\Delta\:}\mathrm{C}\mathrm{t}}={2}^{-\left[\right({\Delta\:}\mathrm{C}\mathrm{t}\:\left(\mathrm{E}\mathrm{x}\mathrm{p}\mathrm{e}\mathrm{r}\mathrm{i}\mathrm{m}\mathrm{e}\mathrm{n}\mathrm{t}\mathrm{a}\mathrm{l}\:\mathrm{g}\mathrm{r}\mathrm{o}\mathrm{u}\mathrm{p}\right)-{\Delta\:}\mathrm{C}\mathrm{t}\:\left(\mathrm{C}\mathrm{o}\mathrm{n}\mathrm{t}\mathrm{r}\mathrm{o}\mathrm{l}\:\mathrm{g}\mathrm{r}\mathrm{o}\mathrm{u}\mathrm{p}\right)]}$$

### Determination of *E. coli* growth at different HMF concentrations

An overnight culture of *E. coli* BL21/pET28a-sumo-*hmfo-vdh*1 was inoculated in a fresh TB medium at 2% (v/v). After VDH1 expression for 12 h, the culture was supplemented with HMF at final concentrations of 0, 10, 20, 30, 40, 50, 60, 70, or 80 mM. *E. coli* BL21/pET28a-sumo-*hmfo-vdh*1 was incubated at 18 °C for 12 h. Growth was monitored by measuring OD_600_ using a spectrophotometer. Samples were taken at 0, 2, 4, 6, 8, 10, and 12 h after HMF addition.

### Analysis of intracellular enzyme expression by SDS-PAGE

Cultured BL21/pET28a-sumo-*hmfo-vdh*1 with 2% inoculum was inoculated in a fresh TB medium (containing 50 µg/mL kanamycin) and cultured until OD_600_ reached 0.8. HMFO and VDH1 expression were analyzed in three experimental groups. In group I, VDH1 was induced by alternating incubation between 42 °C for 0.5 h and 37 °C for 3 h, for a total duration of 12 h. HMFO was continuously induced by adding 0.4 mM IPTG at 18 °C for 12 h. In group Ⅱ, HMFO was induced by adding 0.4 mM IPTG at 18 °C for 12 h. In group Ⅲ, VDH1 was induced by alternating incubation between 42 °C for 0.5 h and 37 °C for 3 h, for a total duration of 12 h. Cells were harvested and broken using an ultrasonic disruptor (Xinzhi, China); crude enzymes were collected for SDS-PAGE analysis.

### Optimization of sequential regulation of dual catalytic modules

The effects of induction time on HMF conversion rate and the effect of transformation temperature on FDCA synthesis in BL21/pET28a-sumo-*hmfo-vdh*1 were investigated using HMF as a substrate. BL21/pET28a-sumo-*hmfo-vdh*1 was cultured overnight at 28 °C and inoculated in a fresh TB medium. When OD_600_ reached ≈ 0.8, VDH1 expression was induced using a temperature alternation strategy. This strategy consisted of cycles alternating between 42 °C for 0.5 h and 37 °C for 3 h. Induction efficacy was assessed after one, two, and three complete cycles, respectively.

After VDH1 expression was induced via the 42 and 37 °C temperature cycle, HMFO expression was induced with 0.4 mM IPTG at 18 °C for 12 h, while simultaneously adding 15 mM HMF. The effects of conversion temperature on FDCA production were analyzed at 25, 30, and 35 °C, with shaking at 200 rpm. Samples were taken at 0, 12, 24, 48, 72, 96, and 120 h. FDCA production was measured using high-performance liquid chromatography (HPLC).

### HPLC analysis

HMF and its oxidized derivatives were analyzed using an Agilent 1260 HPLC system and a UV detector (wavelength = 264 nm). Products were separated and identified using the Aminex HPX-87 H column (300 × 7.8 mm, Bio-Rad, USA) at 60 °C. The mobile phase was 7.5 mM H_2_SO_4_ with a flow rate of 0.6 mL/min; injection volume was 10 µL. The retention times of HMF, HMFCA, DFF, FFCA, and FDCA were 30.79, 21.99, 38.56, 24.46, and 18.46 min, respectively. The concentration (mM) of the oxidized products was calculated from the plotted standard curve. The yield (%) of the oxidized products was calculated using the following formula (2):

2 $$ \:{\mathrm{Yield}}\:\left( \% \right) = \frac{{{\mathrm{Oxidized}}\:{\mathrm{product}}\left( {{\mathrm{mM}}} \right)}}{{{\mathrm{Substrate}}\:{\mathrm{HMF}}\:\left( {{\mathrm{mM}}} \right)}} \times \:100\% $$

### Determination of the thermal stability of VDH1

VDH1 was incubated at 37 and 42 °C, the activity was measured spectrophotometrically at 340 nm by monitoring NADH production, using an extinction coefficient of 6,220 M^− 1^ cm^− 1^.^10^ The reaction mixture (1 mL) contained HMF (10 mM), NAD^+^ (1 mM), and an appropriate amount of enzyme in sodium phosphate buffer (50 mM, pH 7.0). Samples were taken at 0, 3, and 6 h, respectively. All experiments were performed in triplicate.

### Transformation of HMF by dual-enzyme cascade

HMFO and VDH1 were expressed in *E. coli* BL21(DE3) carrying the pET28a-sumo-*hmfo* and pET28a-*vdh*1 plasmids, respectively. HMFO and VDH1 expression was induced by adding 0.4 and 0.1 mM IPTG, respectively; both were incubated at 18 ℃ with shaking at 150 rpm for 12 h. Subsequently, crude enzymes were collected after lysing the cells for protein purification. For protein purification, a nickel-nitrilotriacetic acid column was equilibrated with 50 mM sodium phosphate buffer at pH 7.0. Crude enzymes were loaded into the column and eluted with a linear gradient of imidazole from 20 to 400 mM in 50 mM sodium phosphate buffer containing 0.5 M NaCl. Purified VDH1 and HMFO were used to transform HMF. The dual-enzyme cascade system for HMF conversion was performed in a total volume of 25 mL containing 50 mM sodium phosphate buffer at pH 7.0, 0.1 mM NAD^+^, 10 µM FAD, 0.3 mg/mL HMFO, 0.2 mg/mL VDH1, and 15 mM HMF. The reaction was conducted at 30 °C with shaking at 200 rpm. Samples were taken from the cascade reaction at 0, 12, 24, 48, 72, 96, and 120 h. FDCA production was measured using HPLC.

### Transformation of HMF by the dual-strain catalysis system

BL21/pET28a-sumo-*hmfo* and BL21/pET28a-*vdh*1 were cultured at 18 ℃ for 12 h, and 0.4 mM and 0.1 mM IPTG were added to induce HMFO and VDH1, respectively. Cells were collected at 7000 rpm for 5 min, washed twice with 50 mM sodium phosphate buffer 0 (pH 7.0), and resuspended in 25 mL sodium phosphate buffer, followed by the addition of 15 mM HMF. Reactions were conducted at 30 °C with shaking at 200 rpm for 120 h. Samples were taken at 0, 12, 24, 48, 72, 96, and 120 h. FDCA production was measured using HPLC.

### Statistical analysis

All experiments were performed in three biological replicates, and the data are presented as mean ± SD (standard deviation). Significance analysis was carried out using GraphPad Prism 11. For two-group comparisons, an unpaired two-tailed Student’s *t*-test was used. For multi-group comparisons, one-way ANOVA followed by Tukey’s multiple comparisons test was used. A *pvalue* < 0.05 was considered statistically significant.

### LCA

The LCA methodology consists of four key stages: goal and scope definition, life cycle inventory analysis (LCI), life cycle impact assessment (LCIA), and result interpretation.

Goal and scope: This study performs an LCA of FDCA produced at laboratory scale. The functional unit is a fermentation broth containing 100 g of FDCA (non‑purified). The scope is “gate‑to‑gate”, covering the process from HMF input to FDCA fermentation broth. The system boundary covers the entire HMF‑to‑FDCA conversion, including bacterial culture, induction, and biotransformation, but excludes product separation, raw material transport, and equipment construction. Three synthesis methods (dual‑enzyme cascade, dual‑strain catalysis, and dynamic regulation) are compared. Detailed flows and system boundaries are shown in Table S4‑6 and Fig. S2.

LCI: Inventory data are compiled per functional unit, mainly chemicals and electricity consumption. Data are aggregated from multiple identical small‑scale batches (linear scaling, constant per‑gram impact). Three inventory datasets are established; details are in the Supporting files.

LCIA: LCIA was performed using the SimaPro software based on the Ecoinvent v3.4 database, with environmental impacts evaluated according to the ReCiPe 2016 Midpoint method. The impact categories are listed in Table S7. To facilitate comparison among the three systems, a total ecotoxicity potential (Total ETP) is defined as the sum of the three midpoint categories, including FETP (freshwater), METP (marine), and TETP (terrestrial). All values are expressed in kg 1,4-DCB-eq.

Uncertainty analysis: For each process, the electricity consumption was modeled using a normal distribution where the standard deviation was set to 10% of the mean consumption value. Based on this, a Monte Carlo simulation consisting of 5,000 runs was carried out.

The sensitivity analysis was performed following the methodology and equation proposed in a reported study (Wen et al. 2021).

## Results and discussion

### Construction and verification of sequential regulation of dual catalytic modules

**Fig. 1:**
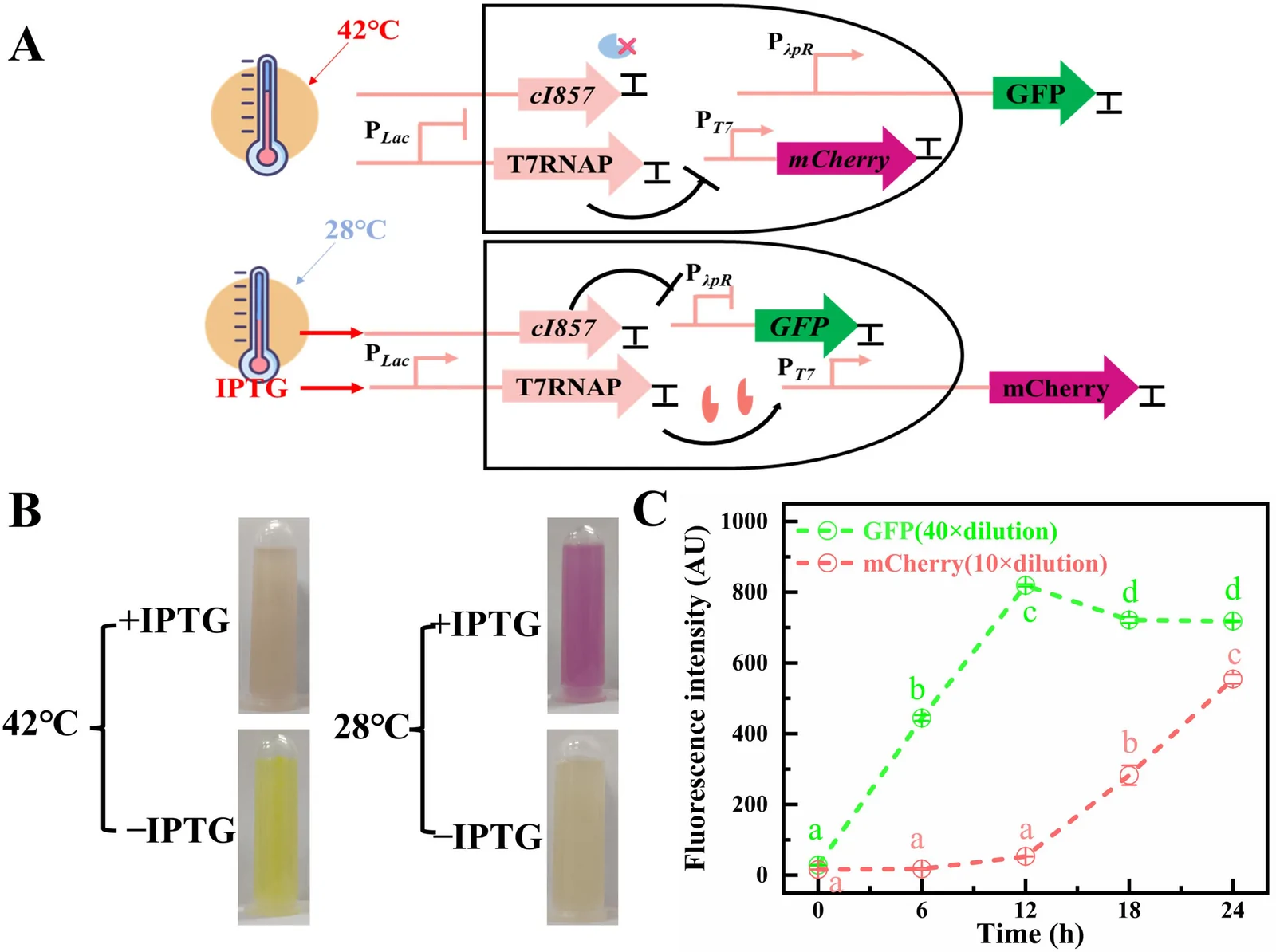
Sequential regulation of dual catalytic modules in the present study. (**A**) Schematic diagram of sequential regulation of dual catalytic modules. (**B**) Sequential regulation of dual catalytic modules expressed GFP and mCherry at different temperatures. (**C**) The fluorescence intensity of mCherry and GFP, and the data are presented as the means ± SD of three independent experiments. Statistical comparisons were performed using one-way ANOVA followed by Tukey’s multiple comparisons test. Different lowercase letters indicate significant differences among groups (*p* < 0.05). Groups sharing the same letter are not significantly different

Sequential regulation of dual catalytic modules was constructed based on the T7 expression system and the λpL/pR-cI857 thermo-inducible system. The strategy was successfully constructed as depicted in Fig. 1. GFP was expressed at 42 °C, whereas mCherry expression was induced by the addition of IPTG at 28 °C (Fig. 1A). To verify the regulatory system, the plasmid containing reporter genes *gfp* and *mcherry* was constructed and transformed into *E. coli* BL21(DE3) to express GFP and mCherry. In the regulatory strain BL21/pET28a-*mcherry-gfp*, mCherry and GFP were expressed successfully at different culture temperatures (Fig. 1B). However, the expression levels of the two fluorescent proteins were critically dependent on the order of temperature shifts (Fig. S3). Specifically, mCherry was robustly expressed at 28 °C; yet, subsequent elevation of the temperature to 42 °C failed to induce GFP expression (Fig. S3). In contrast, when the induction sequence was reversed, GFP was induced first at 42 °C, followed by mCherry expression at 28 °C. Both proteins were successfully and independently expressed (Fig. S3). Accordingly, we sequentially activated the λpL/pR-cI857 thermally inducible expression system prior to induction of the T7 expression system. To further evaluate the BL21/pET28a-*mcherry-gfp* regulatory system, we measured the fluorescence intensities of mCherry and GFP. As shown in Fig. 1C, GFP and mCherry exhibited robust fluorescence intensity after incubation at 42 and 28 ℃, respectively. Initially, both exhibited negligible fluorescence intensities. At 42 ℃ after 6 h, GFP fluorescence intensity was measured at 444.41 ± 7.87 AU and reached its peak after 12 h (819.32 ± 3.31 AU). Subsequently, the T7 expression system was activated to express mCherry by adding IPTG at 28 ℃. mCherry fluorescence intensity increased to 554.32 ± 13.00 AU after 12 h.

The λpL/pR-cI857 thermally inducible system induces gene expression by controlling culture temperature (Li et al. 2024). When the temperature is below 30 °C, the repressor CI857 binds to PL/PR promoter, resulting in transcription cessation. Once the temperature shifts to 42 ℃, the repressor CI857 loses its activity and detaches from the promoter-repressor complex; PL/PR promoter transcription occurs through the action of RNA polymerase (Valdez-Cruz et al. 2010, Valdez-Cruz et al. 2023). However, during thermal induction, prolonged exposure to elevated temperatures inhibits cell growth, affects recombinant protein accumulation, and accompanies heat shock response (Valdez-Cruz et al. 2023). Therefore, a two-stage thermal process is applied: a brief heat shock from 30 to 42 ℃ followed by incubation at 37 ℃, extending the exponential phase and considerably increasing recombinant protein production (Valdez-Cruz et al. 2023). The T7 expression system is widely used for recombinant protein production and for the modular assembly of FDCA synthesis pathways. For example, recombinant *E. coli* harboring *GOase* M3-5, along with HRP and catalase, converts HMF to DFF and 5-formyl-2-furancarboxylic acid (FFCA) with yields of 89.9% and 10.1%, respectively (Tan et al. 2020). Recombinant *E. coli* expressing VDH1 oxidizes HMF to 5-hydroxymethyl-2-furancarboxylic acid (HMFCA) with a yield of approximately 92% (Zhang et al. 2020). Notably, no FDCA production was observed using these approaches. Based on the foregoing analysis, the λpL/pR-cI857 thermally inducible system can be integrated with the T7 expression system to sequentially induce key enzymes by controlling culture temperature, enabling the directed one-pot conversion of HMF to FDCA in a single *E. coli*, thereby reducing by-product accumulation and simplifying the operational process.

### Establishment of sequential regulation of dual catalytic modules for FDCA production

HMFO is a flavin adenine dinucleotide-containing oxidase and belongs to the glucose-methanol-choline-type flavoprotein oxidase family; it is capable of oxidizing HMF to FDCA (Dijkman and Fraaije 2014). However, FFCA conversion to FDCA is the rate-limiting step for HMFO (Vinambres et al. 2020). VDH1 belongs to the aldehyde dehydrogenases family, dependent on coenzymes NAD^+^/NADP^+^, and catalyzes vanillin to vanillic acid (Zhu et al. 2023), with wide applications in biocatalysis and synthetic biology. VDH1 is capable of oxidizing HMF to HMFCA and FFCA to FDCA (Díaz-Maizkurrena et al. 2025, Wang et al. 2020). Consequently, HMFO and VDH1 can be combined to convert HMF to FDCA.

**Fig. 2:**
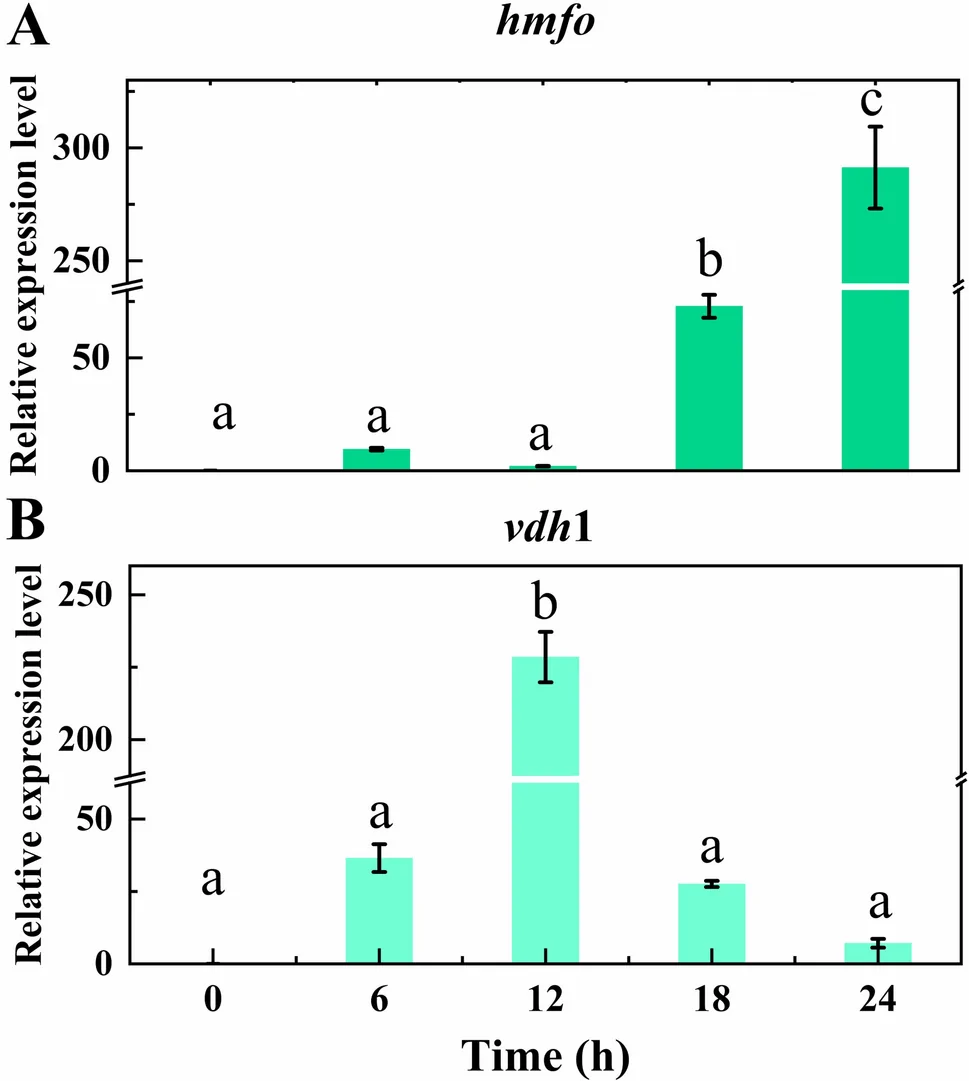
The relative transcription levels of *hmfo* and *vdh*1 in sequential regulation of dual catalytic modules. 16 S rRNA was used as the housekeeping gene for normalization. VDH1 was induced during 0–12 h by alternating incubation between 42 °C for 0.5 h and 37 °C for 3 h. HMFO was induced during 12–24 h by shifting to 18 °C. Data are presented as the means ± SD of three independent experiments. Statistical comparisons were performed using one-way ANOVA followed by Tukey’s multiple comparisons test. Different lowercase letters indicate significant differences among groups (*p* < 0.05). Groups sharing the same letter are not significantly different

In this study, we constructed sequential regulation of dual catalytic modules for HMFO and VDH1 expression to relieve heat damage during thermal induction. VDH1 expression via the 42 and 37 °C cycles and the growth curve of *E. coli* are shown in Fig. S4. The results indicate that temperature cycles between 42 and 37 °C did not substantially affect the growth of the strain BL21/pET28a-sumo-*hmfo-vdh*1. As shown in Fig. S5, HMFO and VDH1 were successfully expressed and analyzed by sodium dodecyl sulfate–polyacrylamide gel electrophoresis (SDS-PAGE). The molecular weights of recombinant HMFO and VDH1 were 68.3 and 50.0 kDa, respectively. On the other hand, the relative transcription levels of *hmfo* and *vdh*1 were analyzed by quantitative polymerase chain reaction (qPCR). As shown in Fig. 2, *hmfo* and *vdh*1 transcription in *E. coli* was upregulated under their respective inducing conditions. Importantly, the thermal inducible system and the T7 system maintained low basal expression under non-inducing conditions. When 42 and 37 °C were alternated for 12 h, the relative transcription levels of *hmfo* and *vdh*1 were 1.99- and 228.5-fold, respectively. After the temperature was adjusted to 18 °C and IPTG was added for 12 h, the relative transcription levels of *hmfo* and *vdh*1 were 291.26- and 7.11-fold, respectively. Hence, the engineered strain BL21/pET28a-sumo-*hmfo-vdh*1 was employed for further experiments.

### Assessment of *E. coli* growth inhibition under varying HMF concentrations

Furfural and HMF are aldehydes whose toxic effects on microbial cells have been reported (Jilani and Olson 2023). To assess the tolerance of *E. coli* BL21/pET28a-sumo-*hmfo-vdh*1 to HMF, we initially analyzed its growth under different HMF concentrations. After VDH1 expression for 12 h, HMF and IPTG were added. As shown in Fig. S6, HMF exhibited strong inhibitory effects on *E. coli* BL21/pET28a-sumo-*hmfo-vdh*1 growth. However, growth was weakly inhibited at HMF concentrations of 0, 10, 20 and 30 mM, with optical density at 600 nm (OD_600_) values reaching approximately 8.81 ± 0.15, 8.20 ± 0.18, 7.75 ± 0.09, and 7.38 ± 0.09, respectively, after cultivation for 12 h. Concentrations exceeding 30 mM slowed growth. At these concentrations, the toxicity of furfural and HMF is primarily attributed to their diffusion across the microbial membrane, which induces multiple cytotoxic effects, including elevated cytoplasmic reactive oxygen species levels, DNA fragmentation, redox imbalance, and inhibition of glycolytic pathway enzymes (Jilani and Olson 2023). Taking into account the growth of *E. coli* and subsequent product yields, 15–25 mM HMF was selected for subsequent experiments.

### Optimization of sequential regulation of dual catalytic modules for FDCA production

**Fig. 3:**
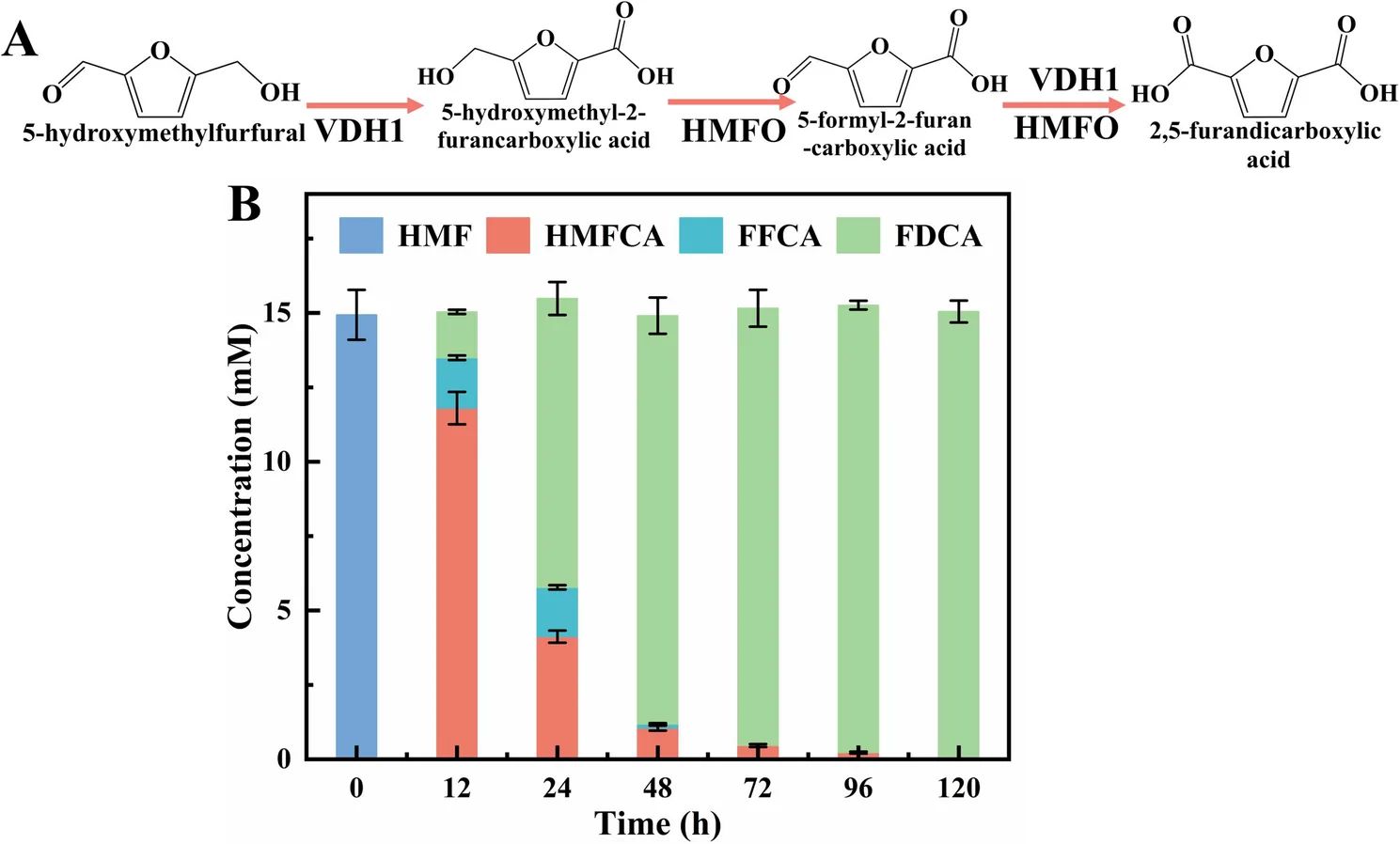
Sequential regulation of dual catalytic modules for FDCA biosynthesis in the present study. (**A**) Sequential regulation of dual catalytic modules pathway for FDCA production. (**B**) Analysis of the FDCA synthesis by sequential regulation of dual catalytic modules using HMF as the substrate. The results were presented as mean ± SD

The stepwise oxidation of HMF to FDCA in our study is illustrated in Fig. 3A. The reaction was divided into two phases. In the first phase, VDH1 was expressed to convert HMF to HMFCA. Subsequently, the culture conditions were regulated to express HMFO in the second phase, where HMFCA was converted to FFCA, and FFCA was further converted with VDH1 to produce FDCA.

To enable biosynthesis of FDCA, we systematically optimized the biotransformation conditions for FDCA production using the engineered strain BL21/pET28a-sumo-*hmfo-vdh*1. As shown in Fig. S7, HMF was completely converted to HMFCA within 1 h. To coordinate expression of the two key enzymes, VDH1 was expressed under an alternating-temperature regimen (42 and 37 °C for 7 h), followed by induction of HMFO expression with IPTG at 18 °C for 12 h. Subsequently, the biotransformation temperature was assessed at 25, 30, and 35 °C. The results indicated that 30 °C was the optimal temperature for FDCA production (Fig. 3B and Fig. S8). At 30 ℃, HMFCA amount decreased to 1.05 ± 0.08 mM, while FDCA amount reached 13.72 ± 0.61 mM at 48 h, indicating that HMFO effectively converted HMFCA to FDCA. After 72 h, FDCA concentration reached 14.70 ± 0.62 mM, corresponding to a yield of 98.0%, with a volumetric productivity of 0.20 mM h^− 1^. The reaction continuously proceeded for 120 h; FDCA concentration reached 15.05 ± 0.37 mM, while the volumetric productivity decreased to 0.13 mM h^− 1^. (Fig. 3B). Meanwhile, the growth curve of *E. coli* BL21/pET28a-sumo-*hmfo-vdh*1 during FDCA biosynthesis was monitored (Fig. S9). The results showed that after 72 h, the cells entered a stationary phase. Under the same conditions, sequential regulation of dual catalytic modules was assessed for FDCA biosynthesis at HMF concentrations of 20 and 25 mM, as shown in Fig. S10. At 20 mM HMF, a maximum FDCA concentration of 17.79 ± 0.58 mM was achieved within 120 h, with a yield of 88.95%, and a volumetric productivity of 0.15 mM h^− 1^. (Fig. S10A). At 25 mM HMF, FDCA reached a maximum concentration of 18.88 ± 0.79 mM within 72 h, with a yield of 75.52%, and a volumetric productivity of 0.26 mM h^− 1^. (Fig. S10B). The thermal stability of recombinant VDH1 was analyzed at 37 and 42 °C, respectively. As shown in Fig. S11, VDH1 exhibited good thermal stability. After 6 h of incubation, VDH1 retained 72.26% and 63.54% of its initial activity at 37 and 42 ℃, respectively.

As previously reported, HMFO is capable of oxidizing HMF to FDCA (Dijkman and Fraaije 2014). Specifically, this study at the 2 mM HMF scale achieves complete conversion using 5 µM HMFO, yielding 92% FFCA and 8% FDCA.^27^ Moreover, when HMFO was combined with lipase, FDCA yield was significantly enhanced to 94.0% (Yuan et al. 2020). Another biocatalytic route toward FDCA is developed by integrating a cell-free extract of GOase variant M3–5 with a whole-cell biocatalyst co-expressing VDHs and NADH oxidase. FDCA was produced with yields exceeding 90% within 36 h at 20 mM HMF (Zhu et al. 2023).

Generally, enzyme catalysis requires enzyme purification, which increases costs. In microbial catalysis, byproduct accumulation and the transmembrane transport of intermediate products are major bottlenecks, thereby restricting the efficient synthesis of FDCA. The sequential regulation of dual catalytic modules in the present study achieved complete conversion of HMF to HMFCA by shifting the temperature to induce VDH1 expression. Subsequently, HMFO expression was induced by adding IPTG, and the reaction continued to convert HMFCA to FFCA. FFCA was further transformed to FDCA by VDH1 and HMFO. This strategy avoided the need for separate cultivation or enzyme purification while decreasing intermediate accumulation, thereby simplifying the overall process. Despite these advantages, the volumetric productivity of the sequentially regulated dual-module strategy remains lower than that of reported FDCA processes with high substrate concentration (Table S1). This limitation may be attributed to the relatively low substrate loading and the prolonged process time required for sequential temperature- and IPTG-induced expression of the two catalytic modules. Further optimization is needed to achieve higher volumetric productivity at higher substrate concentration.

To implement such dynamic regulation, controlled gene expression is crucial. The thermally inducible λpL/pR-cI857 expression system has been widely used to produce recombinant proteins (Riggs 2018, Pawel and Tuck Seng 2015). However, prolonged thermal induction may cause cellular heat stress, inhibiting growth and recombinant protein accumulation (Restrepo-Pineda et al. 2022). To mitigate this, we adopted an alternating-temperature induction strategy (37 and 42 °C); under this optimized strategy, *E. coli* growth remained unaffected, whereas VDH1 demonstrated good thermal stability. Additionally, HMFO exhibited reasonable thermal stability, reflected by a Tm of 48 °C from the denaturation curves (Vinambres et al. 2020). In this study, we observed that FDCA production reached a plateau after 72 h, likely caused by metabolic burden and energy depletion in *E. coli*. (Oftadeh and Hatzimanikatis 2024). In sequential regulation of dual catalytic modules, continuous high-level expression of heterologous proteins, combined with the multi-step oxidation cascade, continuously consumed substantial amounts of ATP, NAD(P)H, and amino acid precursors. Consequently, cells prioritized survival over product synthesis during the late fermentation phase, leading to diminished productivity. To mitigate the metabolic burden and energy depletion, several strategies may be employed in future work to accelerate the transformation from HMF to FDCA, including fed‑batch fermentation (Qu et al. 2013) and cofactor regeneration (Moore et al. 2025).

### FDCA biosynthesis by the dual-enzyme cascade and the dual-strain catalysis system

**Fig. 4:**
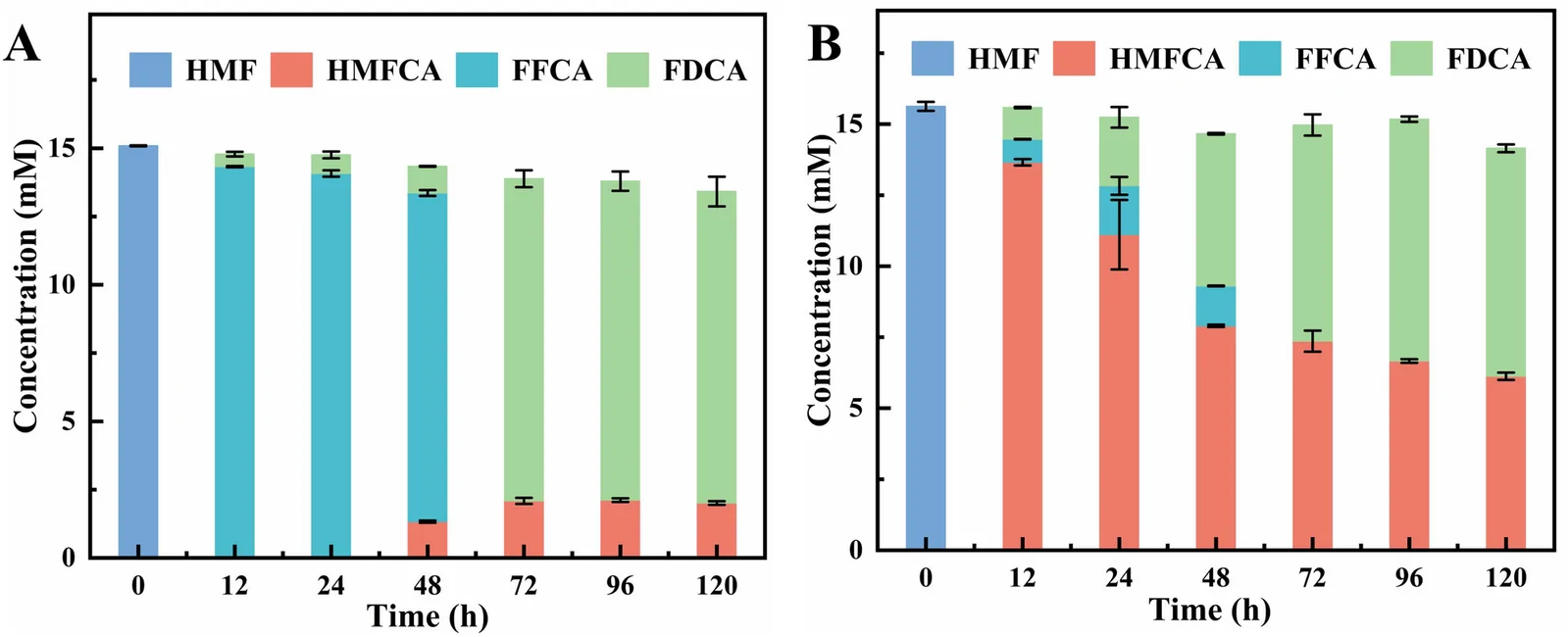
Time-course analysis of FDCA production by the dual-enzyme cascade system and dual-strain catalysis system. (**A**) FDCA was produced by the dual-enzyme cascade system. (**B**) FDCA was produced by the dual-strain catalysis system. The results were presented as mean ± SD

To compare the enzymatic cascade, dual-strain catalysis, and sequential regulation of dual catalytic modules for FDCA synthesis, we further analyzed FDCA production among the dual-enzyme cascade system containing HMFO and VDH1 and the dual-strain catalysis system carrying the *hmfo* and *vdh*1 genes. As shown in Fig. 4, HMF was completely transformed to FDCA by neither the dual-enzyme cascade system nor the dual-strain catalysis system. In the HMFO and VDH1 cascade reaction, HMF was primarily converted to FFCA within the first 12 h. Under these conditions, FFCA concentration was 14.33 ± 0.03 mM. FDCA reached a maximum concentration of 11.79 ± 0.31 mM after 72 h (Fig. 4A). As shown in Fig. 4B, in the dual-strain catalysis system, the substrate was completely transformed within 12 h. HMFCA concentration decreased to 7.9 ± 0.04 mM, whereas FDCA concentration increased to 5.36 ± 0.03 mM after 48 h; FDCA reached a maximum concentration of 8.51 ± 0.09 mM at 96 h.

The results show that, in terms of FDCA synthesis, sequential regulation of dual catalytic modules is more efficient compared with the dual-strain catalysis and dual-enzyme cascade systems. Specifically, when compared separately with the dual-strain and dual-enzyme systems, this system showed 76.4% and 27.2% higher FDCA synthesis efficiency, respectively. Both comparisons were statistically significant (Student’s *t*-test, *p* < 0.0001). The dual-enzyme cascading system requires additional steps for enzyme expression and purification, rendering the process lengthy and economically unfavorable. Free enzyme is highly sensitive to oxidative damage from HMF and the hydrogen peroxide generated by oxidases such as HMFO (Koopman et al. 2010). Whole-cell systems provide a protective intracellular environment that shields biocatalysts from the effects of these reactive substrates. The complex synthetic pathways can be partitioned into two or more microbial strains, with each specializing in a different catalytic step. Nevertheless, in such multi-strain catalysis, the additional transmembrane transport of intermediates between cell populations needed for complete HMF-to-FDCA conversion often limits overall efficiency (Yang et al. 2022). Indeed, transporters for FDCA intermediates in *E. coli* have been reported. For example, AroP, a non-specific HMFCA efflux pump, has been identified, its inactivation significantly reduced the extracellular leakage of HMFCA and consequently increased FDCA titer (Cui et al. 2026). These findings suggest that transport of intermediates may reduce conversion efficiency. In contrast, our single-strain sequential regulation system provides a simpler and more controllable route for the conversion of HMF to FDCA, reducing intercellular intermediate transfer. Moreover, the single-strain system employs sequential expression, which alleviates the metabolic burden associated with concurrent high-level expression of multiple heterologous genes. This mechanism allows the cell to allocate resources to growth and production more efficiently. Given these considerations, sequential regulation of dual catalytic modules emerges as an alternative for FDCA production, primarily owing to its simplicity and dynamic regulation within a single cell, this strategy reduces the need for intermediate transfer between microbial cell populations, although transmembrane transport of intermediates may still occur.

Beyond *E. coli*, an increasing number of microbial systems have been recognized for their advantages in FDCA synthesis. *Bacillus subtilis* J8M8 co‑expressing HmfH, ADH, and MaAAO achieved an FDCA yield of 90.4% (Jiang et al. 2026). A tandem whole‑cell cascade system (*E. coli*_HMFO and *Deinococcus wulumuqiensis* R12) enabled one‑step oxidation of HMF to FDCA with a yield of 99% (Zhang et al. 2024). Both systems possess high substrate tolerance. Building on these advances, future efforts will focus on modular integration of high-tolerance biocatalytic components to further improve FDCA biosynthesis efficiency.

### LCA results

To assess the environmental impacts of producing FDCA from HMF at a laboratory scale (producing a fermentation broth containing 100 g of FDCA, non-purified FDCA products), we analyzed and compared three synthesis strategies, including sequential regulation of dual catalytic modules, dual-strain catalysis, and dual-enzyme cascade systems. Fig. S12 shows the relative contributions of subsystems to environmental impacts. The results showed that step 3 (FDCA production) was the major contributor to environmental impacts in sequential regulation of dual catalytic modules and dual-strain catalysis systems; step 3 was the largest contributor to impacts in 14 of the 18 impact categories. Step 2 (induced expression and protein purification) dominated in the dual-enzyme cascade system and contributed substantially to all impact categories.

**Fig. 5:**
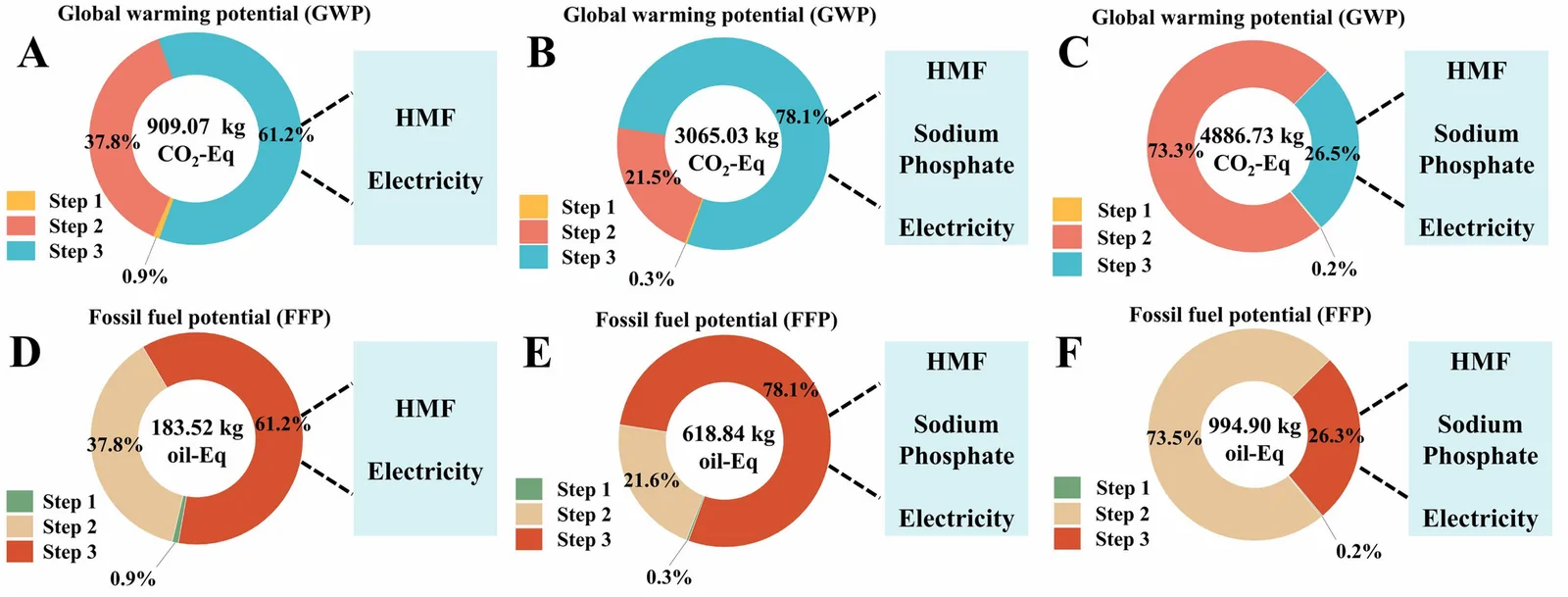
Environmental impacts of the FDCA production on global warming potential and fossil fuel potential. The circle indicated the subsystem and material contributions in sequential regulation of dual catalytic modules, the dual-strain catalysis system, and the dual-enzyme cascade. (A, D) sequential regulation of dual catalytic modules, (B, E) the dual-strain catalysis system, (C, F) the dual-enzyme cascade

Figure 5 presents the environmental impacts of FDCA production, focusing on global warming potential (GWP) and fossil fuel potential (FFP). Total GWP values for sequential regulation of dual catalytic modules, dual-strain catalysis, and dual-enzyme cascade systems were quantified at 909.07, 3065.03, and 4886.73 kg CO₂-eq, respectively (Fig. 5A, B, C). Within these totals, step 3 was the dominant contributor, responsible for 61.2%, 78.1%, and 26.5% of total GWP in the three systems, respectively. Similarly, total FFP values for sequential regulation of dual catalytic modules, dual-strain catalysis, and dual-enzyme cascade systems were 183.52, 618.84, and 994.90 kg oil-eq, respectively. Step 3 contributed substantially, representing 61.2%, 78.1%, and 26.3% of this impact category in each system, respectively (Fig. 5D, E, F). Table 1 presents the environmental impacts of the FDCA production on ecotoxicity potential (ETP), including Freshwater ecotoxicity potential (FETP), Marine ecotoxicity potential (METP) and Terrestrial ecotoxicity potential (TETP). These three midpoint categories were combined into a single “Total ETP” metric because they share the same units (kg 1,4-DCB eq.) under the CML method. This approach is consistent with established LCA report (Andriankaja et al. 2026), in which METP, FETP, and TETP were similarly combined into a single ETP category based on their identical units. Importantly, all disaggregated FETP, METP, and TETP results are also provided separately in Table 1, so the aggregated indicator does not obscure the underlying data. Total ETP for sequential regulation of dual catalytic modules, dual-strain catalysis, and dual-enzyme cascade systems was quantified at 2661.814, 9001.278, and 14694.339 kg 1,4-DCB-eq, respectively (Table 1). Specifically, sequential regulation of dual catalytic modules, dual-strain catalysis, and dual-enzyme cascade systems exhibited lower freshwater ecotoxicity potential (FETP) and marine ecotoxicity potential (METP). FETP values for sequential regulation of dual catalytic modules, dual-strain catalysis, and dual-enzyme cascade systems were 42.90, 144.99, and 230.25 kg 1,4-DCB-eq, respectively, whereas METP values were 56.20, 189.62, and 302.88 kg 1,4-DCB-eq, respectively (Table 1). Based on material contribution, compared with sequential regulation of dual catalytic modules, the dual-strain catalysis and dual-enzyme cascade systems exhibited markedly superior resource consumption and ecotoxicity for FDCA synthesis. The uncertainty analysis results indicate that the rankings of the three systems are robust (Table S8). The sensitivity analysis results for batch duration (Table S9) indicate that the environmental impacts of the dual-strain catalysis system are the most sensitive to batch duration, whereas the dual-enzyme system exhibits the lowest sensitivity. Furthermore, the sensitivity results for chemical inputs (Fig. S13) reveal that across all three systems, tryptone exhibits the most significant positive sensitivity on TETP, while the influence of other chemical inputs remains relatively low. These sensitivity variations and the overall environmental differences are primarily driven by the high consumption of chemical inputs and electricity in the respective systems.

Based on LCA results, sequential regulation of dual catalytic modules demonstrates a lowest environmental impact for FDCA synthesis. The environmental advantage was evident; the dynamic system achieved the lowest GWP (909.07 kg CO₂-eq), FFP (183.52 kg oil-eq), and ETP (2661.814 kg 1,4-DCB-eq), representing reductions of approximately 70–81%, 70–82%, and 70–82%, respectively, against the other two systems. This substantial mitigation of environmental impact is attributed primarily to the system’s integrated design, which simplifies the cellular enrichment steps of the dual-strain catalysis system or enzyme expression and purification required in the dual-enzyme cascade system. Material contribution analysis confirms that electricity consumption is the dominant driver across all systems. Therefore, optimizing this step is most important for further greening the FDCA production process.

Nevertheless, the system boundary in this study does not include upstream HMF production, downstream FDCA separation and purification, which would require additional energy and chemical inputs and generate waste streams. Moreover, the crude FDCA products from the three catalytic systems differ in composition, their purification difficulty and environmental costs may vary, with lower-titer or higher-impurity systems potentially facing greater burdens. The sustainability conclusions are therefore explicitly limited to a laboratory-scale comparative LCA of the catalytic production stage.

**Table 1:** Environmental impacts of the FDCA production on ecotoxicity potential

		Freshwater ecotoxicity potential (FETP) kg 1,4-DCB-eq	Marine ecotoxicity potential (METP) kg 1,4-DCB-eq	Terrestrial ecotoxicity potential (TETP) kg 1,4-DCB-eq	Ecotoxicity potential (ETP) kg 1,4-DCB-eq
Sequential regulation of dual catalytic modules	step1	0.404	0.530	24.543	
	step2	16.642	21.769	1067.486	
	step3	25.855	33.901	1470.684	
	Total	42.90	56.20	2562.71	2661.814
Dual-strain catalysis system	step1	0.780	0.690	71.266	
	step2	32.843	42.897	2259.303	
	step3	111.362	146.028	6336.110	
	Total	144.99	189.62	8666.68	9001.278
Dual-enzyme cascade systems	step1	0.463	0.611	33.782	
	step2	169.416	223.103	10689.576	
	step3	60.371	79.171	3437.846	
	Total	230.25	302.88	14161.20	14694.339

*Total ecotoxicity potential (Total ETP) is defined as the sum of the three midpoint categories, including FETP, METP, and TETP

## Conclusions

In summary, this study described a novel strategy based on the λpL/pR-cI857 thermo-inducible system and the T7 expression system to realize directed one-pot conversion of HMF to FDCA with no detectable accumulation of the measured intermediates and simplifying the operational process. An engineered *E. coli* BL21(DE3)/pET28a-sumo-*hmfo-vdh*1 strain was successfully constructed for FDCA production from HMF by the dynamic regulation of VDH1 and HMFO expression. After optimization, the temperature switch from 42 to 37 ℃ benefited VDH1 expression; 30 ℃ proved optimal for FDCA production. Under the tested conditions, sequential regulation of dual catalytic modules transformed HMF to FDCA with 100% yield compared with the dual-enzyme cascade and dual-strain catalysis systems. LCA revealed that sequential regulation of dual catalytic modules had the lowest environmental impact, particularly in terms of GWP, FFP, and ETP, compared with the dual-enzyme cascade and dual-strain catalysis systems, excluding product separation, transportation, and equipment construction. Sequential regulation of dual catalytic modules established in this study offers a promising platform for developing a directed and sustainable FDCA biosynthesis strategy.

## Supplementary Information

Below is the link to the electronic supplementary material.

Supplementary Material 1

Supplementary Material 2

Supplementary Material 3

## Data Availability

Data and materials will be made available on request.

## Abbreviations

HMFO: Hydroxymethylfurfural oxidase
VDH1: Vanillin dehydrogenase
HMF: 5-hydroxymethylfurfural
FFCA: 5-formyl-2-furancarboxylic acid
HMFCA: 5-hydroxymethyl-2-furancarboxylic acid
DFF: 2,5-dicarboxaldehydefuran
FDCA: 2,5-furandicarboxylic acid
GWP: Global warming potential
FFP: Fossil fuel potential
ETP: Ecotoxicity potential
IPTG: Isopropyl β-D-1-thiogalactopyranoside
LCA: Life cycle assessment
