# Supplementary material for: Sequential regulation of dual catalytic modules for the directed biosynthesis of 2,5-furandicarboxylic acid in Escherichia coli
Source: Bioresour Bioprocess. 2026 Nov 7;13(1):163. doi: 10.1186/s40643-026-01159-5 (PMC13744578; doi:10.1186/s40643-026-01159-5)
Supplement: Supplementary file 1 [file 40643_2026_1159_MOESM2_ESM.pdf]

Cover letter for revision

Dear Editor,

Here we submit our revised manuscript for your consideration in *Bioresources and Bioprocessing*, (entitled: Sequential regulation of dual catalytic modules for the directed biosynthesis of 2,5-furandicarboxylic acid in *Escherichia coli*). The manuscript ID is BIOB-D-26-00444.

We thank the editor and reviewers for their hard work in perfecting our manuscript. We have considered all issues in the comments and answered them point by point. At the same time, we revised the manuscript carefully according to the comments. In addition, we have added the following funding source to the revised manuscript: Open Project Funding of the Key Laboratory of Fermentation Engineering (Ministry of Education) (202609FE04). All changes in the revised manuscript are highlighted in fluorescent green. The responses to the comments point by point are as follows.

We hope that the revised manuscript will be accepted for publication in your esteemed journal. We are looking forward to your reply.

Sincerely,

Zhengbing Jiang

Hubei Key Laboratory of Industrial Biotechnology

Hubei University, Wuhan 430062, People’s Republic of China

E-mail: zhbjiang@hubu.edu.cnReviewer

#1: The authors constructed a sequential-regulation dual-catalytic-module system within a single Escherichia coli cell based on the λpL/pR-cI857 thermally inducible system and the T7 expression system. Biosynthesis of FDCA from 5-HMF was successfully achieved. Based on the analysis of the biosynthetic performance and LCA among the sequential-regulation system, dual-enzyme cascade system and dual-strain catalysis system, the authors propose a promising biomanufacturing strategy for FDCA. The work is systematic, well-designed, and makes significant scientific contributions. Besides it needs some revisions before being accepted by this journal. Some suggestions for revision of this manuscript are listed below.

1. Line 134: The descriptions of plasmids are incomplete. Please supplement the descriptions for each plasmid.

Response: Thank you for your kind comment. Accordingly, we have supplemented the descriptions of each plasmid, optimized the plasmid construction process shown in Fig. S1, and completed Table S2 and Table S3, which list the plasmids, strains and primers.

"The plasmid pDawn-*mcherry* served as the template for *mcherry* gene amplification using primers PET-mCherry-F and PET-mCherry-R; *mcherry* was cloned into linearized pET28a (digested with *Bam*H I, *Not* I) by homologous recombination. The plasmid pET28a-*gfp* was used as the template for *gfp* gene amplification using primers PBV-GFP-F, PBV-GFP-R, and the *gfp* gene was cloned into the linearized pBV220 vector (digested with *Bam*H I, *Eco*R I) to construct the pBV-*gfp*. Subsequently, using pBV-*gfp* as the template, the cI857-gfp-rrnB T1 terminator fragment was amplified with primers T7-CI-F and PET-TER-R and cloned into pET28a-*mcherry* that had been linearized by PCR with primers TER-PET-F and CI-T7-R to construct pET28a-*mcherry-gfp*. The primers used in this study are listed in Table S3; the plasmid construction process is shown in Fig. S1." (Lines 133-139)

Table S2 The plasmids and strains are used in this study

Plasmids and strains	Genotype—descriptions	Sources
Plasmids		
pET28a	P_{T7}	lab stock
pBV220	P_R/P_L	lab stock
pDawn-*mcherry*	Cloning the *mcherry* gene	lab stock
pET28a-*gfp*	Cloning the *gfp* gene	lab stock
pET28a-*mcherry*	Cloning the *mcherry* gene into *Bam*H I and *Not* I sites of pET28a	This study
pBV- *gfp*	Cloning *gfp* gene into *Bam*H I and *Eco*R I sites of pBV220	This study
pET28a-*mcherry-gfp*	One-step cloning *cI857-*P_R/P_L*-gfp-terminator* segment in pET28a-*mcherry*	This study
pET28a-sumo-*hmfo*	*hmfo* gene was synthesized and constructed in pET28a-sumo	This study
pET28a-*vdh*1	*vdh*1 gene was synthesized and constructed in pET28a	This study
pBV- *vdh1*	Cloning *vdh*1 gene into *Bam*H I and *Eco*R I sites of pBV220	This study
pET28a-sumo-*hmfo-vdh*1	One-step cloning *cI857-pR/pL-vdh*1*-terminator* segment in pET28a-sumo-*hmfo*	This study
Strains		
BL21/ pBV- *gfp*	*E. coli* BL21(DE3), P_R/P_L: *gfp*. Kan	This study
BL21/pET28a-*mcherry*	*E. coli* BL21(DE3), P_{T7}: *mcherry*. Kan	This study
BL21/pET28a-*mcherry-gfp*	*E. coli* BL21(DE3), P_{T7}: *mcherry*. P_R/P_L: *gfp*. Kan	This study
BL21/pET28a-sumo-*hmfo*	*E. coli* BL21(DE3), P_{T7}: *hmfo*. Kan	This study
BL21/pET28a-*vdh*1	*E. coli* BL21(DE3), P_{T7}: *vdh*1. Kan	This study
BL21/pET28a-sumo-*hmfo-vdh*1	*E. coli* BL21(DE3), P_{T7}: *hmfo*. P_R/P_L: *vdh*1. Kan	This study

The primers TER-PET-F and CI-T7-R are shown in Table S3.

"Table S3 Primer sequence in this study"

Primer	Sequence
PET-mCherry-F	tggtgtgcggccgcttccttgtacagctc
PET-mCherry-R	aaatgggtcgcggatccatggttagcaaagg
PBV-GFP-F	ttaaggaggaattcatggtgagcaagggc
PBV-GFP-R	cgacggatccccggttatttatacagtt
T7-CI-F	ttcgcgggatcgatcagccaaacgtct
PET-TER-R	gatggcctttttgcgatctcgatcctctac
PBV-VDH-F	attaaggaggaattcatgatcgaacaga
PBV-VDH-R	gacggatccccggttagaacgggta
TER-PET-F	gatggcctttttgcgatctcgatcctctac
CI-T7-R	agacgtttggctgatcgatcccgcgaa
Q-VDH1-F	gtgcggcaacacggttatc

Q-VDH1-R	gagcaacgatagcttccacaac
Q-HMFO-F	cctgccgcgtgattatgatg
Q-HMFO-R	tcagaccagtgacgggaatc
16S-F	catgccgcgtgtatgaagaa
16S-R	cgggtaacgtcaatgagcaaa

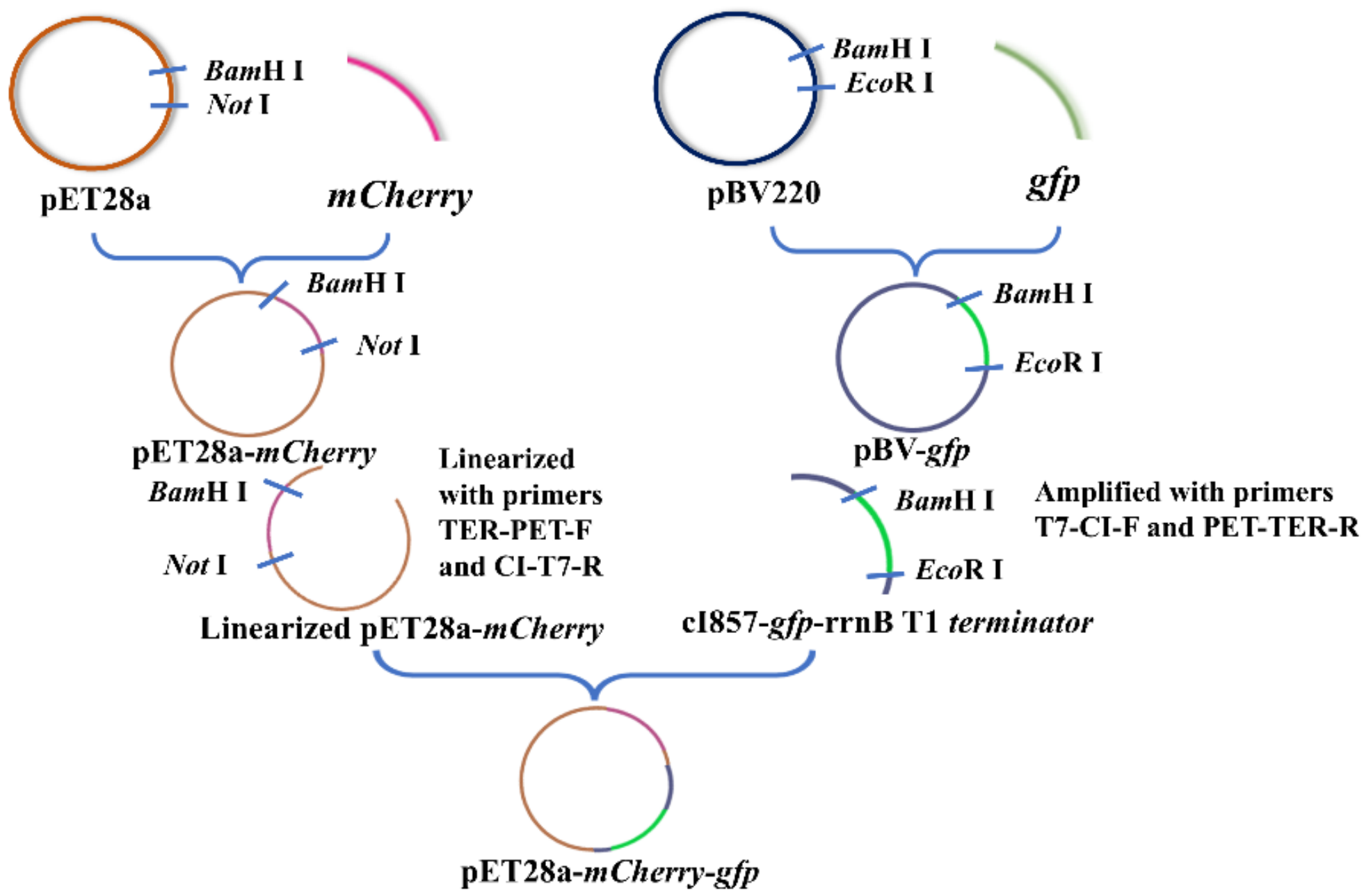

"Fig. S1 The schematic diagram of the plasmid pET28a-mcherry-gfp construction"

2. Line 158: I suggest that the author use the full name instead of abbreviation when qPCR appears for the first time.

Response: Thank you for your kind suggestion. Accordingly, we have provided the full name of qPCR when it first appears.

"Real-time quantitative polymerase chain reaction (qPCR) analysis" (Line 164)

3. Lines 174 and 214: The formatting of formula (1) and (2) requires revision.

Response: Thank you for pointing out this issue. The formatting of formulas (1) and (2) has been revised as follows:

"$2^{-\Delta\Delta Ct} = 2^{-[(\Delta Ct\ (\text{Experimental group}) - \Delta Ct\ (\text{Control group})]}$ (1)" (Line 180)

"$\text{Yield}\ (\%) = \frac{\text{Oxidized product(mM)}}{\text{Substrate HMF (mM)}} \times 100\%$ (2)" (Line 222)

4. Line 208: The formatting of the multiplication sign requires revision.

Response: Thank you for pointing out this issue. We have revised the multiplication sign as follows:

"Products were separated and identified using the Aminex HPX-87H column (300×7.8 mm, Bio-Rad, USA) at 60 °C." (Line 216)

5. The styles of all figures should be unified. For example, the dashed box in Fig. 1 is unnecessary, and labels A and B in Fig. 2 should be consistently formatted.

Response: We appreciate your constructive suggestion. Following your suggestion, we have unified the styles of the figures as follows: Fig. 1 and Fig. 2 have been formatted consistently.

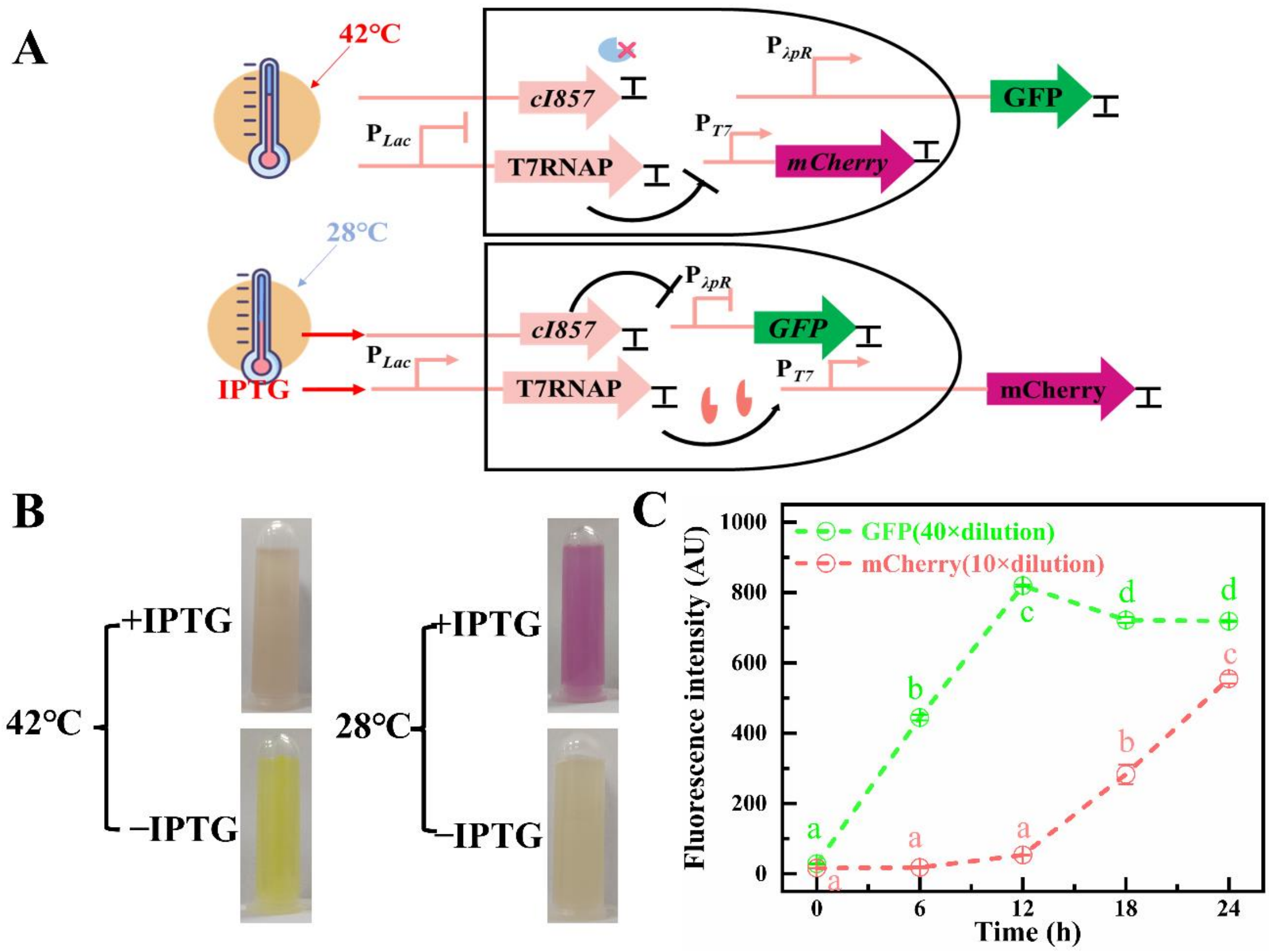

Fig. 1 Sequential regulation of dual catalytic modules in the present study. (A) Schematic diagram of sequential regulation of dual catalytic modules. (B) Sequential regulation of dual catalytic modules expressed GFP and mCherry at different temperatures. (C) The fluorescence intensity of mCherry and GFP, and the data are presented as the means ± SD of three independent experiments. Statistical comparisons were performed using one-way ANOVA followed by Tukey's multiple comparisons test. Different lowercase letters indicate significant differences among groups ($p < 0.05$). Groups sharing the same letter are not significantly different.

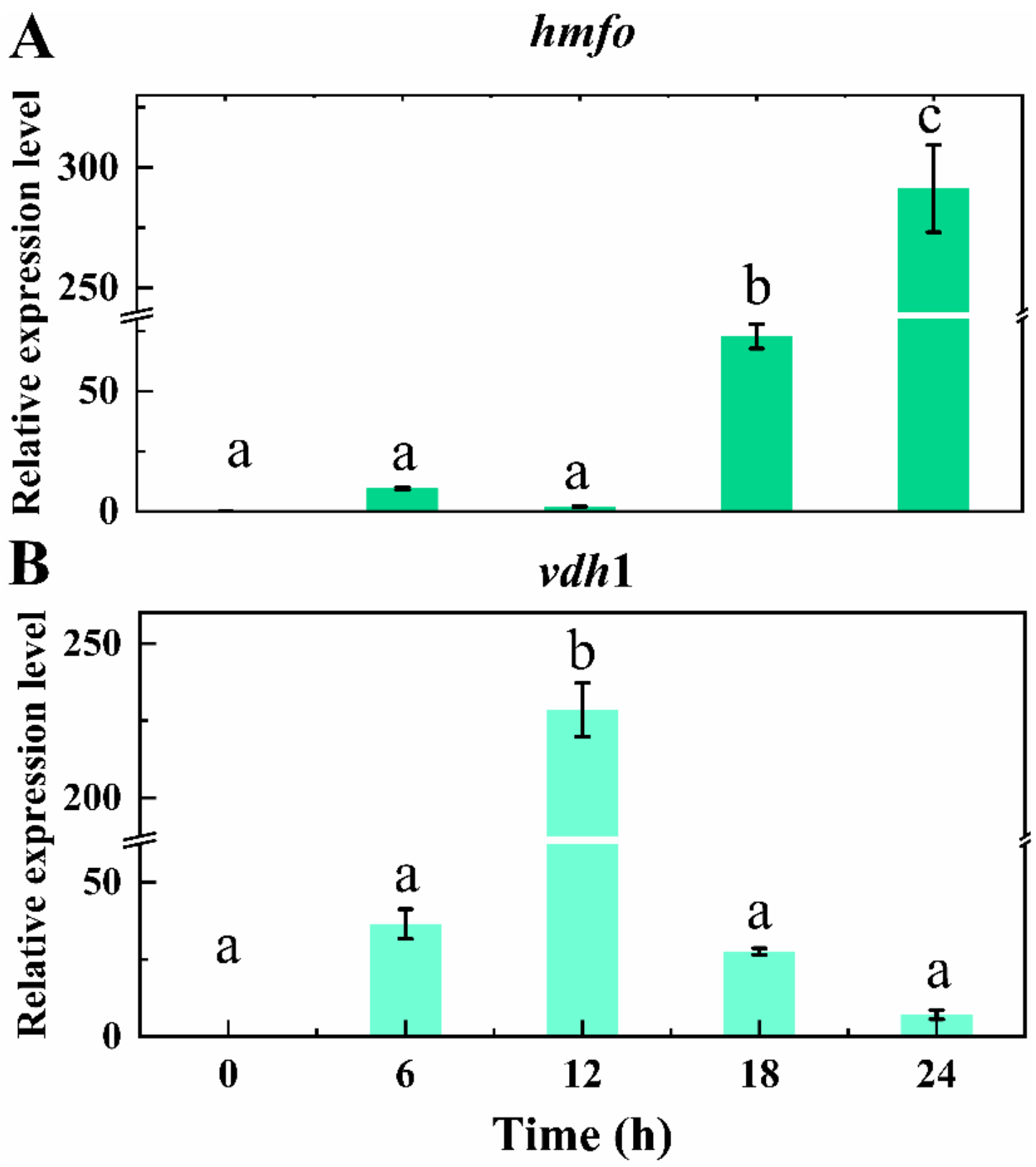

Fig. 2 The relative transcription levels of *hmfo* and *vdh*1 in sequential regulation of dual catalytic modules. 16S rRNA was used as the housekeeping gene for normalization. VDH1 was induced during 0–12 h by alternating incubation between 42 °C for 0.5 h and 37 °C for 3 h. HMFO was induced during 12–24 h by shifting to 18 °C. Data are presented as the means ± SD of three independent experiments. Statistical comparisons were performed using one-way ANOVA followed by Tukey's multiple comparisons test. Different lowercase letters indicate significant differences among groups ($p < 0.05$). Groups sharing the same letter are not significantly different.

6. Line 395: The authors state that the sequential regulation of dual catalytic modules in Escherichia coli can simplify the operational process in FDCA biosynthesis. Please supplemented some discussion about the reported operational process in details.

Response: We appreciate the reviewer's valuable comment. In the revised manuscript, we have supplemented a detailed discussion of the operational process for the sequential regulation of the dual catalytic modules in *E. coli* for FDCA biosynthesis. Meanwhile, we also compared this process with conventional methods and clarified the simplified aspect.

“Generally, enzyme catalysis requires enzyme purification, which increases costs. In microbial catalysis, byproduct accumulation and the transmembrane transport of intermediate products are major bottlenecks, thereby restricting the efficient synthesis of FDCA. The sequential regulation of dual catalytic modules in the present study achieved complete conversion of HMF to HMFCA by shifting the temperature to induce VDH1 expression. Subsequently, HMFO expression was induced by adding IPTG, and the reaction continued to convert HMFCA to FFCA. FFCA was further transformed to FDCA by VDH1 and HMFO. This strategy avoided the need for separate cultivation or enzyme purification while decreasing intermediate accumulation, thereby simplifying the overall process.” (Lines 410-418)

Reviewer #2: The work aims to describe a novel sequential regulatory system in a single Escherichia coli strain to advance the field of FDCA biosynthesis. The approach is novel, and the findings regarding this new strategy are of interest to sustainable biotechnology. In my opinion, this work demonstrates clear scholarly or practical significance, warranting its implementation. However, a better description and discussion of the background and discussion are required to better convey the work's impact. The following comments should be therefore considered before publication.

1. Abstract

-The authors state that microbial catalysis is limited by transmembrane mass transfer efficiency. However, the sequential regulation of dual catalytic modules in E. coli also falls under microbial catalysis.

Response: Thank you for this valuable comment. The single-strain catalysis system in our study is also microbial catalysis. The limitation we referred to applies mainly to multi-strain systems. In multi-strain catalysis system, the intermediates HMFCA and FFCA may transfer between different cells, HMFCA transport into the cytoplasm has been reported to be a rate-limiting step in HMF oxidation and depends on the transporter HmfT1[1]. Although a specific transporter for FFCA has not yet been identified, FFCA is structurally similar to HMFCA, as both contain a carboxylated furan ring. Therefore, its transport likely relies on transporters, which may impose an additional metabolic burden and potentially affect strain stability. Conversely, sequentially regulating dual catalytic modules within a single *E. coli* strain may reduce the possibility of transmembrane transport of intermediates. We have accordingly revised the overly general statement to clarify that this limitation applies mainly to multi-strain catalysis system. The revised statement is as follows:

Reference:

1. Orimaco R, Donnelly P, Sexton S, et al. Characterisation and Harnessing of 5-Hydroxymethylfurfural Metabolism in *Pseudomonas umsongensis* GO16 for the Production of 2,5-Furandicarboxylic Acid. Microbial Biotechnology. 2025 May;18(5):e70159.

“However, enzymatic catalysis is expensive and multi-strain microbial catalysis is limited by the transmembrane transport of intermediates between strains, restricting the efficient synthesis of FDCA and generating a byproduct.” (Lines 19-20)

2. Introduction

2.1 -This sentence requires revision: "Conversely, catalysis with microorganisms and enzymes has also been used in FDCA synthesis".

Response: Thank you for your kind comment. According to your comment, we have revised the sentence. The revised statement is as follows:

“In enzymatic catalysis, complete HMF oxidation often necessitates a combination of two or three enzymes or mediators, combinations such as laccase with (2,2,6,6-tetramethyl-piperidin-1-yl)oxyl,[12] aryl-alcohol oxidase (AAO) with an unspecific peroxygenase,[13] and galactose oxidase (GOase) with lipase have been reported;[14] combinations such as laccase with alcohol oxidase[8] and lipase with alcohol oxidase[15] were reported in our previous study. However, the addition of mediators and purified enzymes in these approaches increases overall cost and environmental impact.[16] Microorganism–enzyme combinations have also been used in FDCA synthesis, such as *Trichoderma reesei* with laccase[17] and *Pseudomonas putida* KT2440 with laccase[18]. Nevertheless, this strategy poses challenges owing to its complex operation and high cost.” (Lines 63-69)

2.2 -Lines 73-79 should be rewritten to clarify the distinction between microbial catalysis and single-strain biosynthesis.

Response: Thank you for this kind suggestion. We have rewritten lines 73–79 to distinguish microbial catalysis and single-strain biosynthesis. The revised statement is as follows:

“Microbial catalysis does not require mediators and enzyme purification. Moreover,

enzymes exhibit superior stability and reduced sensitivity to toxic substrates within the cell,[19] making microbial catalysis a promising strategy for FDCA biosynthesis. Microbial catalysis can be performed using either a single strain or multi-strain catalysis. In multi-strain catalysis, the pathway is divided among different strains. For instance, *E. coli* M overexpressing the *GOase* gene, in combination with *Pseudomonas putida* KT2440,[20] catalyzes the conversion of HMF to FDCA. However, this process is associated with high cost, operational complexity, and inefficient transmembrane transport of intermediates, which limits efficient FDCA synthesis. In single-strain biosynthesis, the requisite enzymes are expressed within a single cell, where the substrate can be efficiently converted to the final product. A previous study reported engineered *Escherichia coli* overexpressing the AAO gene could convert 2,5-dicarboxaldehydefuran (DFF) to FDCA.[16] Although the single-strain biosynthesis of FDCA is characterized by low cost and simple operation, the pathway from HMF to FDCA cannot be precisely directed, which often leads to the accumulation of intermediates. Therefore, a novel FDCA synthesis strategy was constructed to precisely control the pathway, thereby achieving both high catalytic efficiency and complete conversion of HMF to FDCA." (Lines 71-88)

2.3 -Has the sequential regulation of dual catalytic modules been reported previously? If not, how does the authors propose this novel strategy based on which two specific regulatory systems?

Response: Thank you for this insightful question. To the best of our knowledge, the sequential regulation of dual catalytic modules for FDCA synthesis has not been previously reported. Previous studies on FDCA biosynthesis in *E. coli* have mainly

relied on a single inducible system to express enzymes simultaneously, without temporal control over individual catalytic modules. In this work, we established a strategy that combines two independent regulatory systems: the λpL/pR-cI857 thermal inducible system and the T7 expression system. The λpL/pR-cI857 system employs the temperature-sensitive repressor cI857, which binds to the λpL/pR promoters at low temperatures and is inactivated upon a temperature upshift, thereby enabling temperature-dependent gene expression[1,2]. The T7 expression system is widely used owing to its high expression efficiency and strong specificity; transcription is activated by the addition of IPTG.[3] When culture temperature exceeds 37 °C, the binding affinity of T7 RNA polymerase to the P_{T7} promoter weakens,[4] resulting in decreased expression. Therefore, sequential regulation of dual catalytic modules based on the λpL/pR-cI857 thermally inducible system and the T7 expression system can be established in a single strain. By simply adjusting culture temperature and adding IPTG, this strategy can be applied to achieve coordinated control over the expression of two or more genes involved in converting HMF to FDCA.

Reference:

1. Li, F.; Chen, Q.; Deng, H.; Ye, S.; Chen, R.; Keasling, J. D.; Luo, X., One-pot selective biosynthesis of Tyrian purple in *Escherichia coli*. *Metab Eng* **2024,** *81*, 100-109. https://doi.org/10.1016/j.ymben.2023.11.003.

2. Valdez-Cruz, N. A.; Caspeta, L.; Pérez, N. O.; Ramírez, O. T.; Trujillo-Roldán, M. A., Production of recombinant proteins in *E. coli* by the heat inducible expression system based on the phage lambda pL and/or pR promoters. *Microbial Cell Factories* **2010,** *9* (1), 18. https://doi.org/10.1186/1475-2859-9-18.

3. Lin, T. H.; Cheng, S. Y.; Lin, Y. F.; Chen, P. T., Development of the Low-Temperature Inducible System for Recombinant Protein Production in *Escherichia coli* Nissle 1917. *J Agric Food Chem* **2024,** *72* (13), 7318-7325. https://doi.org/10.1021/acs.jafc.4c01075.

4. Boulain, J. C.; Dassa, J.; Mesta, L.; Savatier, A.; Costa, N.; Muller, B. H.; L'Hostis, G.; Stura, E. A.; Troesch, A.; Ducancel, F., Mutants with higher stability and specific activity from a single thermosensitive variant of T7 RNA polymerase.

Protein Eng Des Sel **2013,** *26* (11), 725-34. https://doi.org/10.1093/protein/gzt040.

3. Results and Discussion

3.1 -I suggest that the author label the subgraphs in Figure 2 as A and B, respectively.

Response: Thank you for kind suggestion. Following your suggestion, we have revised Fig. 2 as follows:

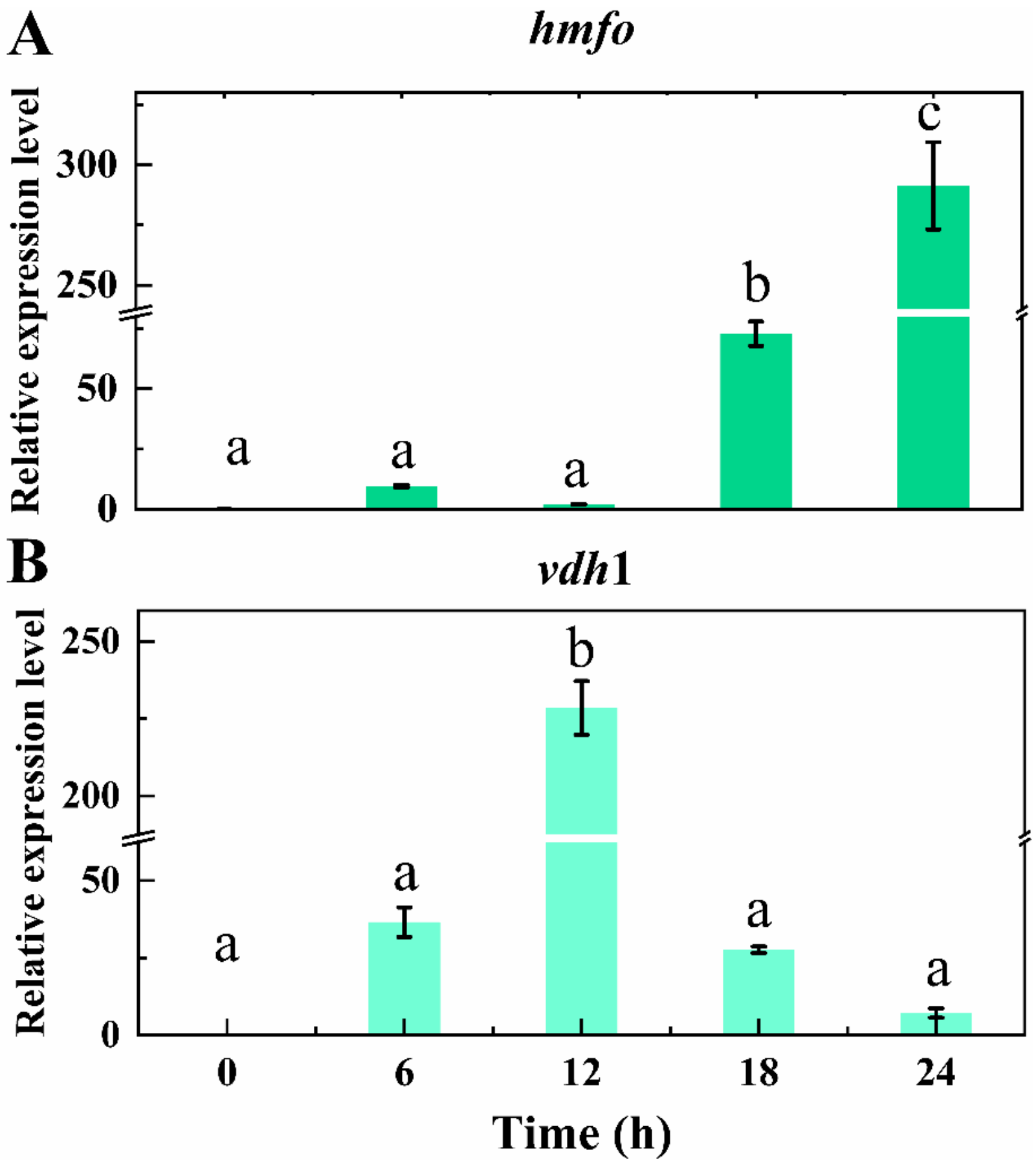

Fig. 2 The relative transcription levels of *hmfo* and *vdh*1 in sequential regulation of dual catalytic modules. 16S rRNA was used as the housekeeping gene for normalization. VDH1 was induced during 0–12 h by alternating incubation between 42 °C for 0.5 h and 37 °C for 3 h. HMFO was induced during 12–24 h by shifting to 18 °C. Data are presented as the means ± SD of three independent experiments. Statistical comparisons were performed using one-way ANOVA followed by Tukey's multiple comparisons test. Different lowercase letters indicate significant differences among groups ($p < 0.05$). Groups sharing the same letter are not significantly different.

3.2 -The authors state that the dual-strain catalysis relies on transmembrane mass transfer to complete full transformation from substrate to FDCA. Is there literature

reporting potential transmenbrane transporter for the intermediates of FDCA in E. coli? Please discuss it to strengthen the advantage of sequential regulatory system operating within a single strain.

Response: We appreciate your valuable comment. Indeed, transporters for FDCA intermediates in *E. coli* have been reported. We have discussed transmembrane transporters in the revised manuscript. The statement is as follows:

"Nevertheless, in such multi-strain catalysis, the additional transmembrane transport of intermediates between cell populations needed for complete HMF-to-FDCA conversion often limits overall efficiency.[49] Indeed, transporters for FDCA intermediates in *E. coli* have been reported. For example, AroP, a non-specific HMFCA efflux pump, has been identified, its inactivation significantly reduced the extracellular leakage of HMFCA and consequently increased FDCA titer.[50] These findings suggest that transport of intermediates may reduce conversion efficiency. In contrast, our single-strain sequential regulation system provides a simpler and more controllable route for the conversion of HMF to FDCA, reducing intercellular intermediate transfer. Moreover, the single-strain system employs time-series expression, which alleviates the metabolic burden associated with concurrent high-level expression of multiple heterologous genes. This mechanism allows the cell to allocate resources to growth and production with higher specificity." (Lines 467-476)

Reference:

49. Cui Y, Zhang P, Miao R, et al. Multi-dimensional metabolic engineering and enzyme engineering in *Escherichia coli* for highly efficient biosynthesis of 2, 5-furandicarboxylic acid[J]. Bioresource Technology, 2026: 134989.

Reviewer #3: The manuscript develops a two-stage expression strategy that combines the λpL/pR-cI857 thermo-inducible system with the T7 expression system to sequentially control VDH1 and HMFO expression in Escherichia coli for HMF-to-FDCA conversion. The single-strain concept is potentially useful, and the comparison with dual-enzyme and dual-strain systems is of interest. However, several issues related to the causal role of sequential regulation, quantitative comparability of the catalytic systems, analytical mass balance, and the interpretation of the LCA should be addressed before the conclusions can be fully supported.

1. The central claim is that sequential regulation of the two catalytic modules itself improves directed FDCA biosynthesis. However, the current comparisons are mainly against a purified dual-enzyme cascade and a dual-strain system, which simultaneously change catalyst format, mass-transfer behavior, enzyme loading, and process operation. A matched single-strain control in which hmfo and vdh1 are expressed simultaneously under otherwise identical cultivation, biomass, and biotransformation conditions is needed to isolate the benefit of sequential expression. A reverse-order induction control would also be informative. Without such controls, the improvement cannot be attributed specifically to sequential regulation.

Response: Thanks the reviewer for this rigorous and important comment. We provided the matched single-strain control, BL21/pCDFDuet-sumo-*hmfo*-*vdh*1, which was processed under identical cultivation, biomass, and biotransformation conditions. BL21/pCDFDuet-sumo-*hmfo*-*vdh*1 and BL21/pET28a-sumo-*hmfo*-*vdh*1 were adjusted to an initial OD600 of 0.8, with 0 h defined as the time of HMF addition. The growth curve of BL21/pET28a-sumo-*hmfo*-*vdh*1 is shown in Fig. S9. The growth and HMF conversion of BL21/pCDFDuet-sumo-*hmfo*-*vdh*1 are shown in Fig. R1 below. FDCA reached a maximum of 2.22 ± 0.06 mM by 120 h, with a yield of 14.80%, while biomass began to decline after 48 h. In addition, the results of the reverse-order induction control are shown in Fig. S3. This control failed to achieve sequential regulation, resulting in a low FDCA titer of 1.37 mM; therefore, the corresponding production data are not presented.

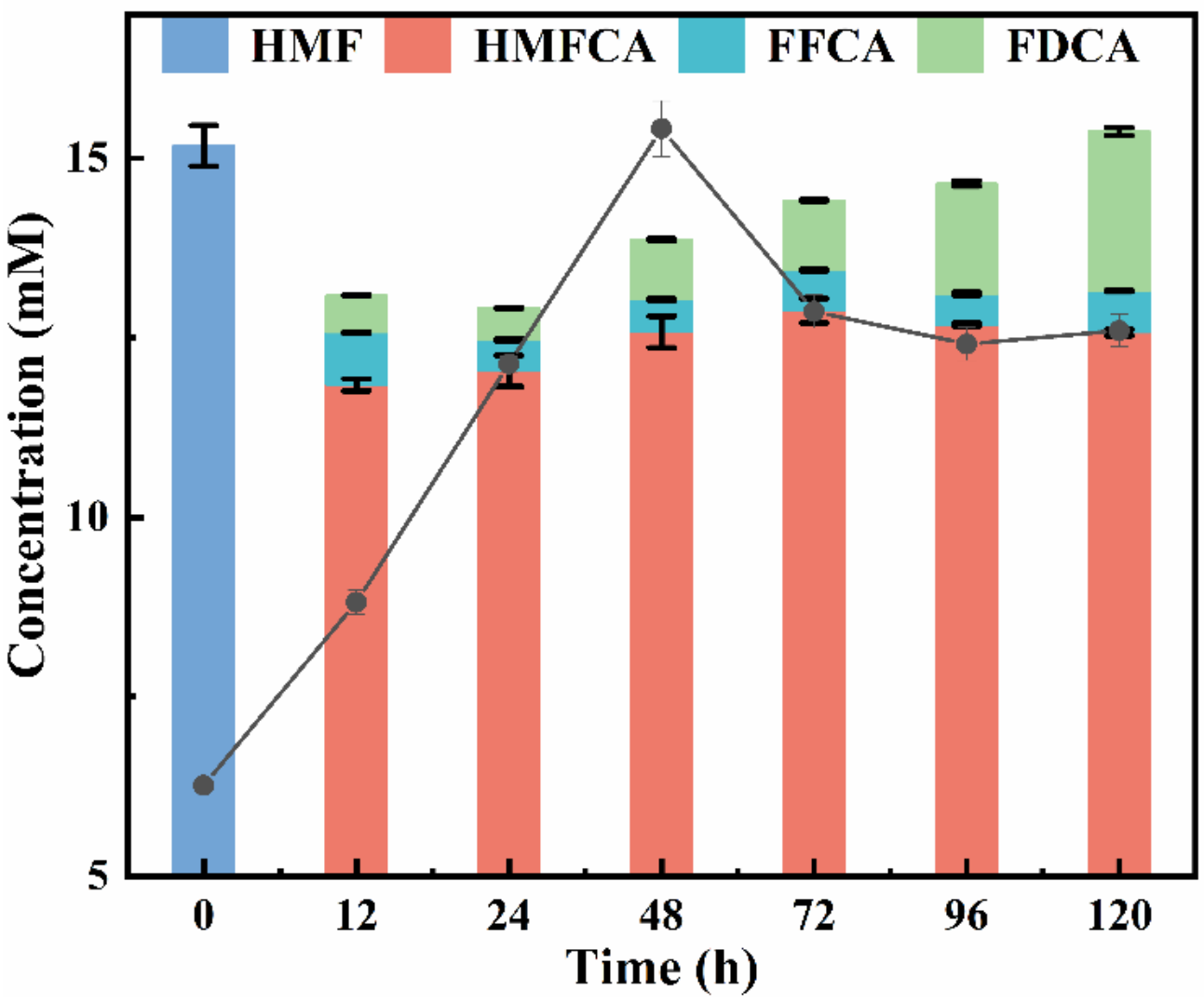

Fig. R1 The growth and HMF conversion of BL21/pCDFDuet-sumo-*hmfo*-*vdh*1

2. The three catalytic systems should be compared on an equivalent quantitative basis. The dual-enzyme experiment reports defined HMFO and VDH1 concentrations, whereas the methods for the dual-strain and sequential single-strain systems do not clearly state the biomass loading, OD600/dry cell weight, cell ratio, total cellular protein, or effective enzyme activities used in the reactions. Please report and normalize these parameters. Otherwise, the differences in FDCA yield may partly reflect unequal catalyst loading rather than the architecture of the catalytic system.

Response: Thanks for pointing out these problems. We clarify that, for the dual-enzyme and dual-strain systems, BL21/pET28a-sumo-*hmfo* and BL21/pET28a-*vdh*1 were each cultured in duplicate under identical conditions (four flasks in total). For each strain, one flask was used for the preparation of purified enzymes for the dual-enzyme system, and the other was used for the dual-strain system. For the dual-strain and sequential single-strain systems, the initial OD_{600} for cultivation was 0.8. At the start of FDCA production, the OD_{600} values were 7.63±0.30 and 7.26±0.54, respectively.

3. The manuscript repeatedly describes the system as providing "real-time" and "precise" dynamic regulation, but the evidence currently consists mainly of qPCR and qualitative SDS-PAGE measurements after externally imposed temperature/IPTG switches. Please quantify the temporal protein abundance and/or catalytic activity of

VDH1 and HMFO and characterize basal expression/leakiness of both modules. There are also inconsistencies in the reported induction schedule: the qPCR methods state sampling at 0, 12, and 24 h, whereas Fig. 2 contains 0, 6, 12, 18, and 24 h data; three cycles of 42 °C for 0.5 h and 37 °C for 3 h total 10.5 h, although several passages refer to 12 h; and the optimized VDH1 induction is later described as 7 h. Please reconcile these details throughout the Methods, figure legends, and Results

Response: Thanks for pointing out these problems. Prior to constructing the dual catalytic modules, we separately evaluated the conversion capabilities of VDH1 and HMFO towards HMF, DFF, HMFCA, and FFCA. The results are shown in Fig. R2 and Fig. R3. VDH1 converted HMF to HMFCA and FFCA to FDCA. HMFO converted HMF to DFF and FFCA (producing a minor amount of FDCA), DFF and HMFCA to FFCA (with minor FDCA production), and FFCA to FDCA. Based on these specific conversion characteristics of VDH1 and HMFO towards HMF and its derivatives, we constructed a dual catalytic module. In this system, VDH1 was expressed to convert HMF to HMFCA. Subsequently, the culture conditions were regulated to express HMFO in the second phase, where HMFCA was converted to FFCA, and FFCA was further converted with VDH1 to produce FDCA. As shown in Fig. S7, when VDH1 was induced for 3.5, 7 and 10.5 h, 15 mM HMF was completely converted to HMFCA within 1 h, Under these conditions, the FFCA concentrations were 0.70±0.11, 2.51±0.13, and 1.74±0.04 mM, respectively, while the FDCA concentrations were 0.43±0.02, 0.60±0.05, and 0.52±0.02 mM, respectively. As shown in Fig. 3B, when HMFO was induced for 12 h, the FFCA and FDCA concentrations reached 3.87±0.24 mM and 1.91±0.12 mM, respectively. Therefore, HMFO has a very low level of basal expression without induction. Compared with individual VDH1 and HMFO, our dual catalytic module exhibited significantly higher efficiency. We apologize for the oversight regarding the induction schedule in our original manuscript. We have now clearly added and described the detailed induction schedule in the revised manuscript. The statements are as follows:

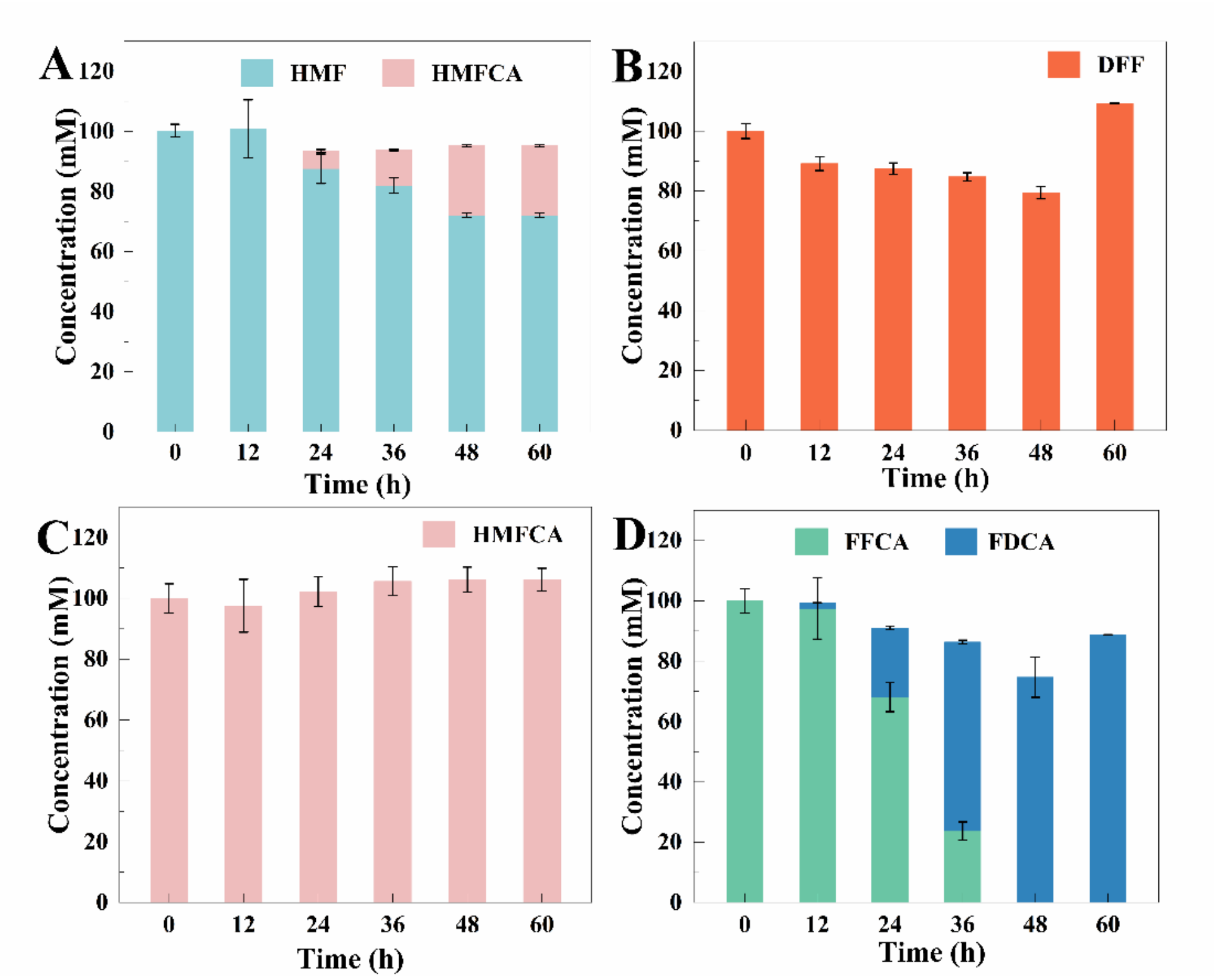

Fig. R2 Conversion of HMF and its derivatives by VDH1. A. HMF, B. DFF, C. HMFCA, D. FFCA

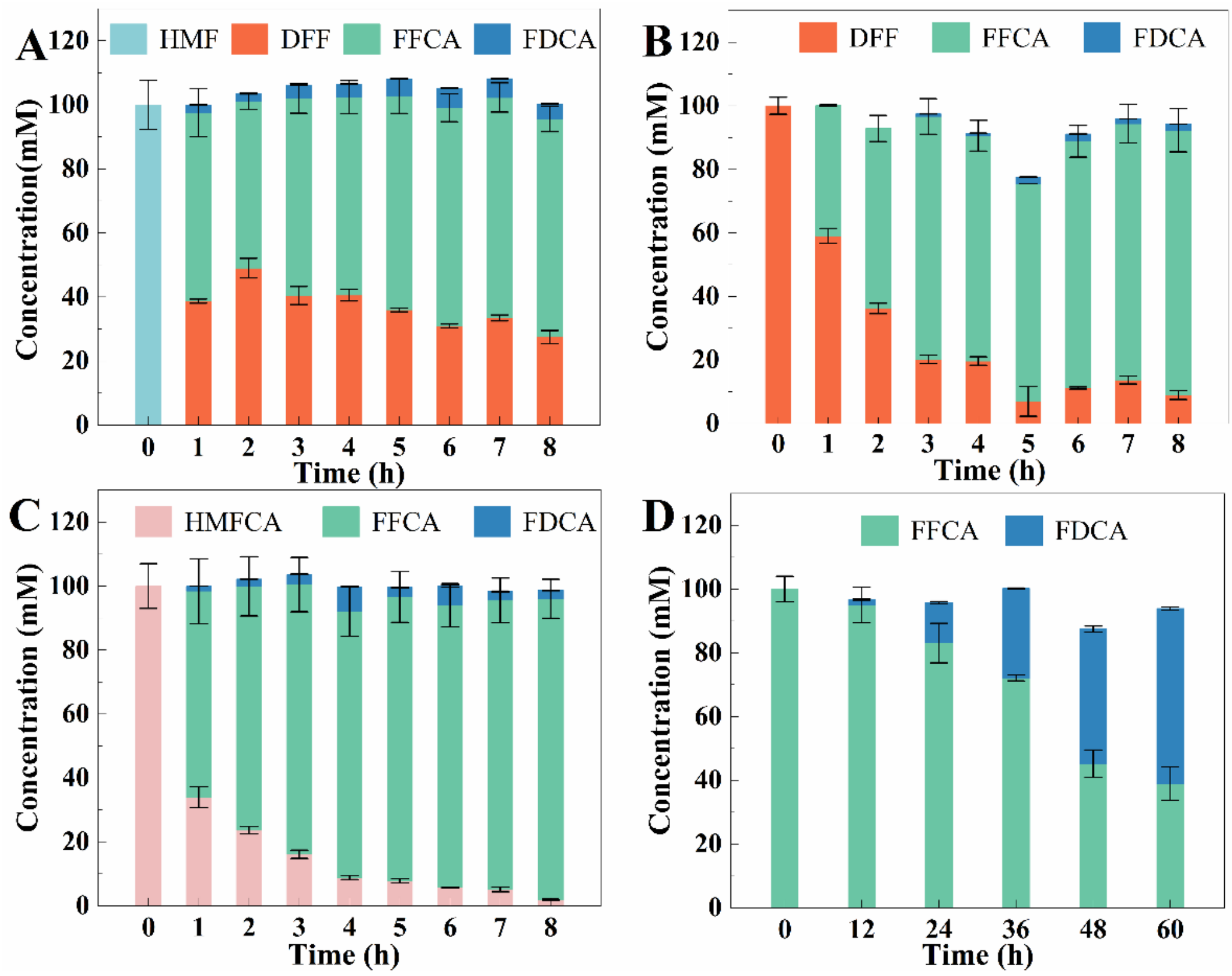

Fig. R3 Conversion of HMF and its derivatives by HMFO. A. HMF, B. DFF, C. HMFCA, D. FFCA

In the "**Experimental section**":

"VDH1 was induced by alternating incubation between 42 °C for 0.5 h and 37 °C for 3 h, for a total duration of 12 h. Later, HMFO was induced by adding 0.4 mM IPTG at 18 °C for 12 h. BL21/pET28a-sumo-*hmfo-vdh*1 was cultured at 30 °C as the control group; samples were taken to analyze the relative transcription levels of *hmfo* and *vdh*1 at 0, 6, 12, 18 and 24 h." (Lines 170-174)

"In group I, VDH1 was induced by alternating incubation between 42 °C for 0.5 h and 37 °C for 3 h, for a total duration of 12 h. HMFO was continuously induced by adding 0.4 mM IPTG at 18 °C for 12 h. In group II, HMFO was induced by adding 0.4 mM IPTG at 18 °C for 12 h. In group III, VDH1 was induced by alternating incubation between 42 °C for 0.5 h and 37 °C for 3 h, for a total duration of 12 h." (Lines 192-196)

In the "**Figure legend**":

"Fig. 2 VDH1 was induced during 0–12 h by alternating incubation between 42 °C for 0.5 h and 37 °C for 3 h."

"Fig. S4 The growth curve of *E. coli* BL21/pET28a-sumo-*hmfo-vdh*1 under different culture temperatures. *E. coli* was cultured at 37°C for the untreated control, while the experimental group underwent alternating incubation at 42 °C for 0.5 h and 37 °C for 3 h, for a total duration of 12 h."

4. The analytical evidence for "100% yield" and "no byproduct accumulation" should be strengthened. The HPLC method states that HMF, HMFCA, DFF, FFCA, and FDCA were quantified, yet DFF is not shown in the main time-course figures. Please report all measured pathway species, provide detection/quantification limits, and present a molar or carbon balance over time. At 15 mM initial HMF, the reported FDCA concentration of 15.05 ± 0.37 mM is slightly above the starting substrate concentration; please explain how yield values were calculated and handled within analytical error. Unless broader product analysis was performed, the wording should be limited to "no

detectable accumulation of the measured pathway intermediates" rather than a general claim of no byproducts.

Response: We thank the reviewer for this rigorous and important comment. Because HMFO can convert HMF to DFF[1], we included a method for DFF detection. However, no DFF was detected in the actual reactions, it is possible DFF was further converted to FFCA. The limit of detection (LOD) and limit of quantification (LOQ) for the HPLC method were determined using the residual standard deviation method. The LOD was 0.000114 mM and the LOQ was 0.000345 mM (with a standard error of ±0.000058). The original data for all measured pathway species, including the intermediate products in the pathway, are shown in Fig. R4 below. As shown in the data, the initial HMF concentration was 15.04±0.56 mM. After 120 h, the FDCA concentration reached 15.05 ± 0.37 mM. Although the final FDCA concentration is numerically slightly higher than the initial HMF concentration, the difference (0.01 mM) is entirely within the standard deviation of the measurements. This indicates a complete 1:1 molar conversion of HMF to FDCA, with a carbon balance of approximately 100%. In addition, we have revised the description in the manuscript, replacing the general claim of "no byproducts" with the more precise statement. The statements are as follows:

Time/h	Production	peak area			Product concentration/mM			Average	Stdev.p
0	HMF	6934.622	7542.323	7023.346	14.56	15.83	14.74	15.04	0.56
12	FDCA	1459.39	1662.454	1686.221	1.74	1.98	2.01	1.91	0.12
	HMFCA	3877.048	4400.357	4411.609	6.60	7.49	7.51	7.20	0.42
	FFCA	1739.1	1979.314	1986.425	3.54	4.04	4.05	3.87	0.24
24	FDCA	7449.727	8154.96	8556.241	8.98	9.83	10.32	9.71	0.55
	HMFCA	2267.787	2444.743	2557.874	3.86	4.16	4.35	4.12	0.20
	FFCA	796.807	832.736	881.142	1.57	1.65	1.75	1.66	0.07
48	FDCA	11988.06	11381.14	10753.52	14.46	13.73	12.97	13.72	0.61
	HMFCA	687.636	588.984	578.115	1.16	1.00	0.98	1.05	0.08
	FFCA	128.383	95.223	98.656	0.18	0.11	0.12	0.14	0.03
72	FDCA	11800.08	11837.09	12901.47	14.24	14.28	15.57	14.70	0.62
	HMFCA	256.703	252.926	315.506	0.43	0.42	0.53	0.46	0.05
96	FDCA	12340.72	12586.05	13956.91	14.89	15.19	16.84	15.04	0.15
	HMFCA	162.21	119.379	125.834	0.27	0.20	0.21	0.22	0.03
120	FDCA	13020.6	13836.37	13433.17	14.37	15.27	14.82	15.05	0.37

Fig. R4 The original data on FDCA biosynthesis at 15 mM HMF scale

Reference:

1. Dijkman, W. P.; Fraaije, M. W., Discovery and characterization of a 5-

hydroxymethylfurfural oxidase from Methylovorus sp. strain MP688. Appl Environ Microbiol 2014, 80 (3), 1082-90.

In the "**Abstract**" section:

"In this study, we aimed to establish sequential regulation of dual catalytic modules in Escherichia coli to precisely control the expression of hydroxymethylfurfural oxidase (HMFO) and vanillin dehydrogenase (VDH1), enabling the directed one-pot conversion of HMF to FDCA with no detectable accumulation of the measured intermediates and simplifying the operational process." (Lines 24-25)

"Overall, this study establishes a more efficient and sustainable strategy for directed FDCA biosynthesis." (Lines 34-35)

In the "**Conclusions**" section:

"In summary, this study described a novel strategy based on the λpL/pR-cI857 thermo-inducible system and the T7 expression system to realize directed one-pot conversion of HMF to FDCA with no detectable accumulation of the measured intermediates and simplifying the operational process." (Line 569)

"Under the tested conditions, sequential regulation of dual catalytic modules transformed HMF to FDCA with 100% yield compared with the dual-enzyme cascade and dual-strain catalysis systems." (Line 575)

5. Please recheck the percentage comparison in the discussion of Fig. 4. The statement that FDCA production was 43.3% and 21.4% "higher" than the dual-strain and dual-enzyme systems appears to use the sequential-regulation value as the denominator. If the intention is to report relative increases over the comparator systems, the denominator should be the comparator value; alternatively, report the absolute percentage-point differences in yield. The same convention should be used consistently in the Abstract, Results, and Conclusions.

Response: We thank the reviewer for pointing out this issue and apologize for this oversight. All percentage comparisons have been carefully rechecked, and the relevant statements in the Abstract, Results, and Discussion sections have been revised accordingly.

"Specifically, when compared separately with the dual-strain and dual-enzyme systems, this system showed 76.4% and 27.2% higher FDCA synthesis efficiency, respectively. Both comparisons were statistically significant (Student's *t*-test, $p < 0.0001$)." (Lines 458-460)

6. The biochemical description of VDH1 should be corrected. The manuscript states that VDH1 is an NAD+/NADP+-dependent aldehyde dehydrogenase but also states that it converts vanillin to vanillyl alcohol. This is inconsistent with an aldehyde dehydrogenase reaction and with the NADH-production assay described in the Methods. Please verify the correct product (presumably vanillic acid), and check all reaction arrows and enzyme annotations in the text and figures.

Response: We thank you for pointing out this important inconsistency. We have carefully rechecked the relevant report on the biochemical function of VDH1, corrected this error throughout the manuscript, and checked all reaction arrows and enzyme annotations in the text and figures. The revised statement is as follows:

"VDH1 belongs to the aldehyde dehydrogenases family, dependent on coenzymes NAD^+/$NADP^+$, and catalyzes vanillin to vanillic acid,[28] with wide applications in biocatalysis and synthetic biology." (Line 334)

7. The practical performance at higher substrate loading requires a more balanced discussion. Although the 15 mM reaction approaches quantitative FDCA formation, the yield decreases to 88.95% at 20 mM and 75.52% at 25 mM, with reaction times of 72-120 h. Please report volumetric productivity (for example, g L−1 h−1 or mmol L−1 h−1) and compare it with relevant whole-cell and cascade processes, rather than relying

on final yield alone. Claims that the strategy overcomes the bottleneck of whole-cell FDCA production or is readily applicable to industrial production should be tempered unless higher substrate loadings and process productivity are demonstrated.

Response: We thank you for this constructive comment. We have recalculated the volumetric productivity for the three systems in the revised manuscript. We also compared these values with representative whole-cell and enzymatic cascade systems reported in the literature (Additional Table S1). We have revised the claims that the strategy overcomes the bottleneck of whole-cell FDCA production or is readily applicable to industrial production. The statements are as follows:

In the "**Abstract**" section:

"Overall, this study establishes a more efficient and sustainable strategy for directed FDCA biosynthesis." (Lines 34-35)

In the "**Results and Discussion**" section:

"After 72 h, FDCA concentration reached 14.70±0.62 mM, corresponding to a yield of 98.0%, with a volumetric productivity of 0.20 mM h^{-1}. The reaction continuously proceeded for 120 h; FDCA concentration reached 15.05±0.37 mM, while the volumetric productivity decreased to 0.13 mM h^{-1}. (Fig. 3B)." (Lines 388-390)

"At 20 mM HMF, a maximum FDCA concentration of 17.79±0.58 mM was achieved within 120 h, with a yield of 88.95%, and a volumetric productivity of 0.15 mM h^{-1}. (Fig. S10A). At 25 mM HMF, FDCA reached a maximum concentration of 18.88±0.79 mM within 72 h, with a yield of 75.52%, and a volumetric productivity of 0.26 mM h^{-1}. (Fig. S10B)." (Lines 396-399)

"This strategy avoided the need for separate cultivation or enzyme purification while decreasing intermediate accumulation, thereby simplifying the overall process. Despite

these advantages, the volumetric productivity of the sequentially regulated dual-module strategy remains lower than that of reported FDCA processes with high substrate concentration (Table S1). This limitation may be attributed to the relatively low substrate loading and the prolonged process time required for sequential temperature- and IPTG-induced expression of the two catalytic modules. Further optimization is needed to achieve higher volumetric productivity at higher substrate concentration."

(Lines 417-424)

8. The HMF-tolerance experiment was performed with BL21/pET28a in LB at 37 °C, whereas the production strain is BL21/pET28a-sumo-hmfo-vdh1, cultivated in TB and subjected to repeated 42/37 °C shifts followed by 18 °C induction. Because heterologous-protein burden and thermal cycling can substantially alter stress tolerance, please either assess HMF tolerance and cell viability/growth using the actual production strain under the production conditions or provide a clear justification for transferring the tolerance result from the control strain.

Response: We thank the reviewer for this rigorous and important comment. Accordingly, we have supplemented experiments to assess the tolerance of the production strain to varying HMF concentrations under production conditions, and the results are shown in figure below. In addition, we have revised the methods and description of *E. coli* growth inhibition under varying HMF concentrations. The statements are as follows:

In the "**Experimental section**":

"An overnight culture of *E. coli* BL21/pET28a-sumo-*hmfo-vdh*1 was inoculated in a fresh TB medium at 2% (v/v). After VDH1 expression for 12 h, the culture was supplemented with HMF at final concentrations of 0, 10, 20, 30, 40, 50, 60, 70, or 80 mM. *E. coli* BL21/pET28a-sumo-*hmfo-vdh*1 was incubated at 18 °C for 12 h. Growth

was monitored by measuring OD_{600} using a spectrophotometer. Samples were taken at 0, 2, 4, 6, 8, 10, 12 h after HMF addition." (Lines 182-187)

In the "**Results and Discussion"** section:

"To assess the tolerance of *E. coli* BL21/pET28a-sumo-*hmfo-vdh*1 to HMF, we initially analyzed its growth under different HMF concentrations. After VDH1 expression for 12 h, HMF and IPTG were added simultaneously. As shown in Fig. S6, HMF exhibited strong inhibitory effects on *E. coli* BL21/pET28a-sumo-*hmfo-vdh*1 growth. However, growth was weakly inhibited at HMF concentrations of 0, 10, 20 and 30 mM, with optical density at 600 nm (OD_{600}) values reaching approximately 8.81±0.15, 8.20±0.18, 7.75±0.09, and 7.38±0.09, respectively, after cultivation for 12 h. Concentrations exceeding 30 mM slowed growth." (Lines 359-366)

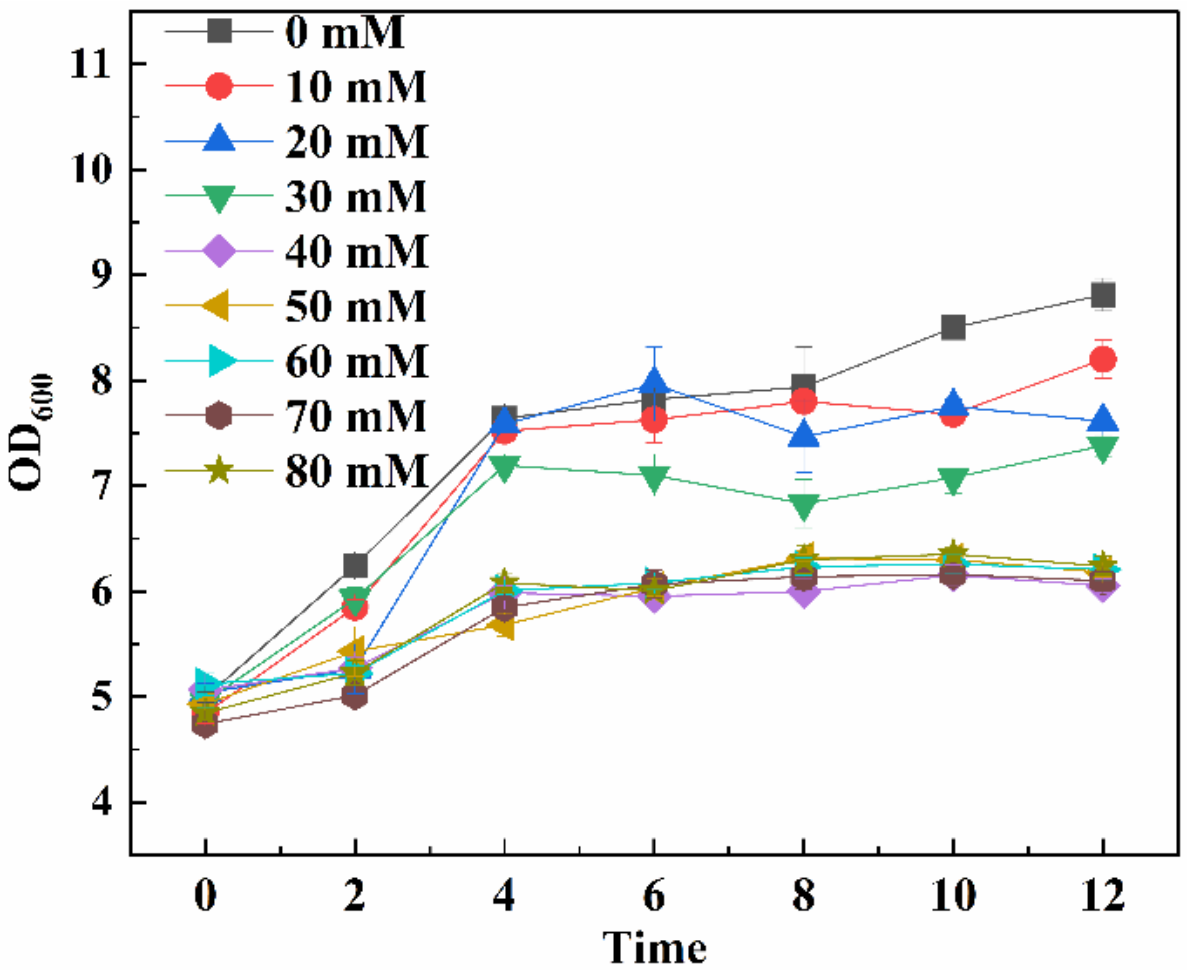

Fig. S6 The growth curve of *E. coli* BL21/pET28a under different HMF concentrations

9. The proposed mass-transfer explanation is stronger than the presented evidence. A single-strain pathway can reduce the need for intermediate transfer between different cell populations, but it does not eliminate transmembrane transport of HMF, FDCA, or potentially some intermediates. No direct transport measurements are presented. Please rephrase statements such as "radically avoids the transmembrane mass transfer limitations" and distinguish reduced intercellular intermediate transfer from general

membrane-transport limitations.

Response: We thank the reviewer for this important point. Accordingly, we have revised the relevant description of intercellular intermediate transfer and membrane-transport limitations, and we have rephrased statements such as “radically avoids the transmembrane mass-transfer limitations”. The statements are as follows:

In the “**Abstract**” section:

“However, enzymatic catalysis is expensive and multi-strain microbial catalysis is limited by the transmembrane transport of intermediates between strains, restricting the efficient synthesis of FDCA and generating a byproduct.” (Lines 19-20)

“However, this process is associated with high cost, operational complexity, and inefficient transmembrane transport of intermediates, which limits efficient FDCA synthesis.” (Lines 78-79)

In the “**Results and Discussion”** section:

“Generally, enzyme catalysis requires enzyme purification, which increases costs. In microbial catalysis, byproduct accumulation and the transmembrane transport of intermediate products are major bottlenecks, thereby restricting the efficient synthesis of FDCA.” (Lines 410-413)

“Nevertheless, in such a multi-strain configuration, the additional transmembrane transport of intermediates between cell populations needed for complete HMF-to-FDCA conversion often limits overall efficiency.[49]” (Lines 467-469)

“Moreover, the single-strain system employs sequential expression, which alleviates the metabolic burden associated with concurrent high-level expression of multiple

heterologous genes. This mechanism allows the cell to allocate resources to growth and production more efficiently. Given these considerations, sequential regulation of dual catalytic modules emerges as an alternative for FDCA production, primarily owing to its simplicity and dynamic regulation within a single strain, this strategy reduces the need for intermediate transfer between microbial cell populations, although transmembrane transport of intermediates may still occur." (Lines476-483)

10. The LCA system boundary and functional unit require clearer justification. The study is described as "cradle-to-gate", but the stated boundary begins with the HMF-to-FDCA conversion and excludes downstream separation/purification, transport, and equipment construction. Please clarify exactly which upstream burdens of HMF, media components, inducers, cofactors, and electricity are included. In addition, the functional unit is a fermentation broth containing 100 g of non-purified FDCA; because the three systems produce broths with different concentrations and impurity profiles, please report the corresponding broth volumes/compositions and discuss how this choice affects the comparison.

Response: We thank the reviewer for this important comment. Following ISO 14040:2006 and the goal and scope of LCA[1,2], we have revised the system boundary and functional unit accordingly. The analysis starts with HMF as the input and ends with the FDCA fermentation broth. For HMF, media components, inducer, cofactors, and other chemicals, only their direct consumption at the fermentation step was accounted for; their upstream production was excluded. Transport, equipment construction, and downstream purification were also excluded. Therefore, we now refer to it as a "gate-to-gate" LCA, covering the process from HMF input to FDCA fermentation broth. The functional unit is defined as "Fermentation broth containing 100g FDCA". The Life Cycle Inventory (LCI) for FDCA production includes media components, inducer, cofactors, other chemicals, and electricity, as listed in the Supplementary LCA inventory (Tables S4–S6). Because the three systems have different FDCA titers, the input inventories for the FDCA production step were normalized to the fermentation broth volume required to produce 100 g FDCA. The

sequential regulation of dual catalytic modules, dual-strain catalysis systems, and the dual-enzyme system produced FDCA titers of 2.34 g/L, 1.33 g/L, and 1.84 g/L, corresponding to fermentation broth volumes of 46.69 L, 75.30 L, and 54.35 L, respectively. The major components of the fermentation broths are listed in Table R1 below. The current functional unit does not consider downstream burdens, which may lead to an underestimation of the actual environmental impacts of systems with lower titers or higher impurity loads, thereby biasing the comparison in favor of these systems. In addition, the primary goal of our study is to identify the environmental hotspots of different biocatalysis systems on the production-stage processes, so the residual substrates, intermediates, byproducts, salts, and cell biomass are output streams rather than inputs. Because the system boundary ends at fermentation and excludes downstream purification and waste treatment, these output streams are not included in the input-side impact assessment. We have added a discussion that if downstream purification or waste treatment were included, systems with lower titers or higher impurity loads could incur additional burdens. The statements are as follows:

Table R1 The major components of the fermentation broth.

	Component	Fermentation broth volumes
Sequential regulation of dual catalytic modules	HMF, Tryptone, Yeast Extract, Glycerol, KH_2PO_4, K_2HPO_4, Kanamycin, IPTG, Water	46.69 L
The dual-strain catalysis systems	HMF, Na_2HPO_4, $NaH_2PO_4 \cdot 2H_20$, Water	75.30 L
The dual-enzyme system	HMF, Na_2HPO_4, $NaH_2PO_4 \cdot 2H_20$, Water	54.35 L

Reference:

1. Curran M A. Overview of goal and scope definition in life cycle assessment[M]//Goal and scope definition in life cycle assessment. Dordrecht: Springer Netherlands, 2016: 1-62.
2. Doyle L, Borgogno C, Cavero G. Report on LCA and LCC: Baseline, scope, functional units definition and database review social media presence[J].

In the “**Experimental section”:**

“The functional unit is a fermentation broth containing 100 g of FDCA (non-purified). The scope is “gate-to-gate”, covering the process from HMF input to FDCA fermentation broth” (Line 263)

In the “**Results and Discussion”** section:

“Nevertheless, the system boundary in this study does not include upstream HMF production, downstream FDCA separation and purification, which would require additional energy and chemical inputs and generate waste streams. Moreover, the crude FDCA products from the three catalytic systems differ in composition, their purification difficulty and environmental costs may vary, with lower-titer or higher-impurity systems potentially facing greater burdens. The sustainability conclusions are therefore explicitly limited to a laboratory-scale comparative LCA of the catalytic production stage.” (Lines 556-562)

11. The LCA is based on linear scaling of laboratory-scale batches with a constant per-gram impact, while electricity is reported as the dominant contributor. Such an assumption can strongly magnify laboratory heating, shaking, centrifugation, and purification energy and may not represent scale-up behavior. The current uncertainty analysis varies only electricity consumption by ±10%. Please expand the sensitivity/uncertainty analysis to include major process variables such as yield, batch duration, biomass/enzyme production, chemical inputs, and electricity scenario, and explain the regional electricity mix used. The manuscript should also justify the summation of freshwater, marine, and terrestrial ecotoxicity midpoint indicators into a single "Total ETP" metric, or report these categories separately. The sustainability conclusion should be framed explicitly as a laboratory-scale comparative LCA of the

catalytic production stage.

Response: We thank the reviewer for this rigorous and important comment. Laboratory-scale batch data can overestimate the electricity demand of scale-sensitive unit operations, including heating, shaking/agitation, centrifugation, and purification. Accordingly, the revised manuscript expands the sensitivity analysis to include batch duration, chemical inputs, and electricity supply scenarios, and clarifies the electricity mix assumed. The sensitivity coefficient for chemical inputs is presented in Figure S13, the sensitivity coefficients for batch duration are presented in Table S9. In SimaPro modeling, we selected the Ecoinvent dataset "market group for electricity, low voltage CN" to represent the Chinese regional low-voltage electricity supply. The software's interpretation of this data is as follows: “This dataset is a market group. Market groups represent a ‘market of markets’. Their purpose is to group a set of markets into larger, geographically relevant datasets.” For a more detailed power structure, we also conducted an extended analysis of this data from the software. As shown in Fig. R5, the market group is divided into the China Southern Power Grid (CSG, ~17.75%) and the State Grid Corporation of China (SGCC, ~82.25%). The SGCC is further subdivided into five regional grids: East China (ECGC, ~29.9%), North China (NCGC, ~23.7%), Northwest (NWG, ~16.4%), Northeast (NECG, ~15.3%), and Central China (CCG, ~14.7%). There is no detailed presentation or explanation of the more detailed power structure within the software. In addition, we have justified aggregating the METP, FETP, and TETP midpoint categories into a single “Total ETP” metric and added a limitation statement clarifying that the sustainability conclusion is limited to a laboratory-scale comparative LCA of the catalytic production stage. The statements are as follows:

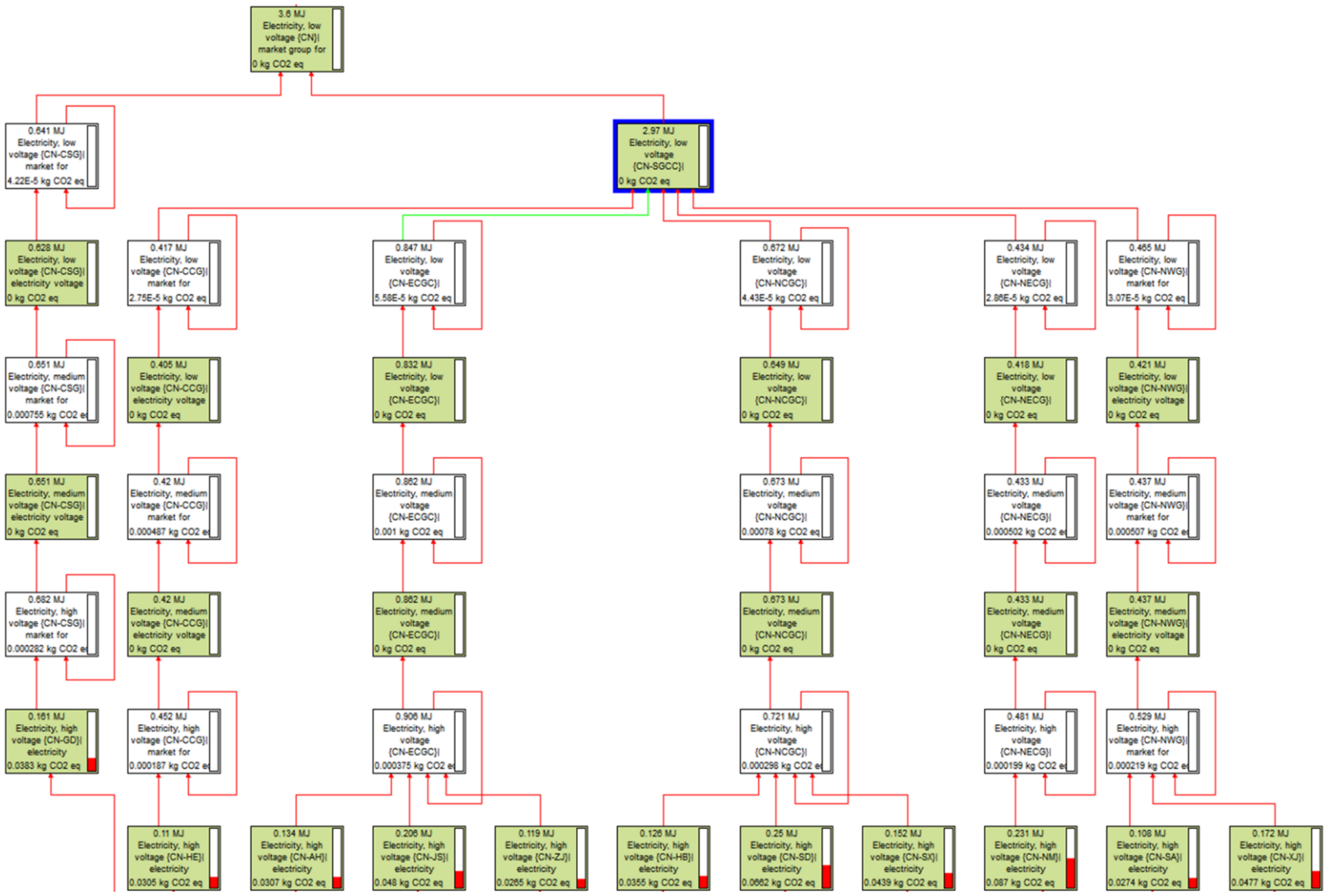

Fig. R5 Detailed breakdown of the "market group for electricity, low voltage CN" in ecoinvent, showing the shares of regional grids

In the "**Experimental section**":

"The sensitivity analysis was performed following the methodology and equation proposed in a reported study.[32]" (Lines 282-283)

In the "**Results and Discussion**" section:

"Table 1 presents the environmental impacts of FDCA production on ecotoxicity potential (ETP), including freshwater ecotoxicity potential (FETP), marine ecotoxicity potential (METP), and terrestrial ecotoxicity potential (TETP). These three midpoint categories were combined into a single "Total ETP" metric because they share the same units (kg 1,4-DCB eq.) under the CML method. This approach is consistent with

established LCA report [53], in which METP, FETP, and TETP were similarly combined into a single ETP category based on their identical units. Importantly, all disaggregated FETP, METP, and TETP results are also provided separately in Table 1, so the aggregated indicator does not obscure the underlying data. Total ETP for sequential regulation of dual catalytic modules, dual-strain catalysis, and dual-enzyme cascade systems was quantified at 2661.814, 9001.278, and 14694.339 kg 1,4-DCB-eq, respectively (Table 1)." (Lines 517-522)

"The uncertainty analysis results indicate that the rankings of the three systems are robust (Table S8). The sensitivity analysis results for batch duration (Table S9) indicate that the environmental impacts of the dual-strain catalysis system are the most sensitive to batch duration, whereas the dual-enzyme system exhibits the lowest sensitivity. Furthermore, the sensitivity results for chemical inputs (Fig. S13) reveal that across all three systems, tryptone exhibits the most significant positive sensitivity on TETP, while the influence of other chemical inputs remains relatively low. These sensitivity variations and the overall environmental differences are primarily driven by the high consumption of chemical input and electricity in the respective systems." (Lines 534-540)

"Moreover, the crude FDCA products from the three catalytic systems differ in composition, their purification difficulty and environmental costs may vary, with lower-titer or higher-impurity systems potentially facing greater burdens. The sustainability conclusions are therefore explicitly limited to a laboratory-scale comparative LCA of the catalytic production stage." (Lines 559-562)

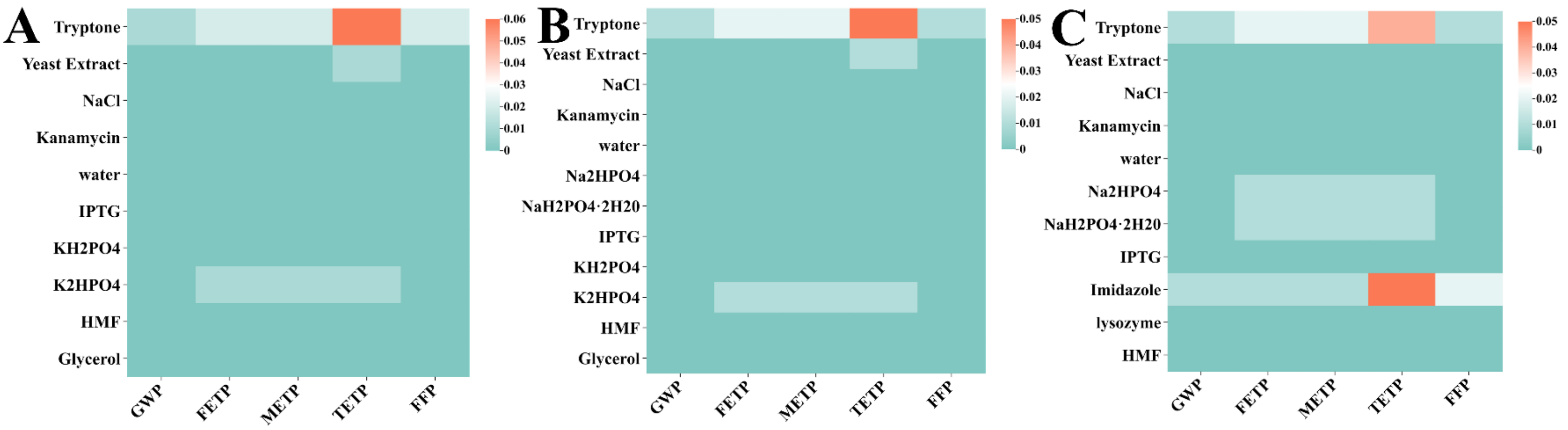

Fig. S13 Sensitivity coefficient of LCA to chemical inputs. A. Sequential regulation of dual catalytic modules, B. The dual-strain catalysis systems, C. The dual-enzyme system

Table S9 Sensitivity coefficient of LCA to batch duration

	GWP	FETP	METP	TETP	FFP
Sequential regulation of dual catalytic modules	0.61	0.60	0.60	0.57	0.61
The dual-strain catalysis systems	0.78	0.77	0.77	0.73	0.78
The dual-enzyme system	0.27	0.26	0.26	0.24	0.26

Reference:

32. Wen, Z.; Xie, Y.; Chen, M.; Dinga, C. D., China's plastic import ban increases prospects of environmental impact mitigation of plastic waste trade flow worldwide. Nature Communications 2021, 12 (1), 425.

52. Andriankaja H, Penot J D, Chaudron R, et al. A comparative environmental assessment of an automotive component processed by laser powder bed fusion (LPBF) versus CNC machining, with steel powder reuse impact analysis[J]. Cleaner Engineering and Technology, 2025: 101137.

12. Please strengthen the statistical reporting and overall presentation. The Methods state that Student's t-test or one-way ANOVA was used, but it is not clear which test was used for each comparison, whether post-hoc multiple-comparison tests were

applied, or what exact P values support statements such as "significantly higher" in the Abstract. Figure legends should state n, the statistical test, and significance notation where appropriate. The figures would also benefit from higher-resolution labels and more consistent terminology. Several typographical and grammatical issues should be corrected throughout (for example, "dual-stain" versus "dual-strain", "higher efficient", and "Menawhile").

Response: We sincerely thank the reviewer for this constructive and important comment. We have carefully revised the methods, statistical reporting, figures, figure legends, and typographical and grammatical issues. The statements are as follows:

In the "**Experimental section**":

"All experiments were performed in three biological replicates, and the data are presented as mean±SD (standard deviation). Significance analysis was carried out using GraphPad Prism 11. For two-group comparisons, an unpaired two-tailed Student's *t*-test was used. For multi-group comparisons, one-way ANOVA followed by Tukey's multiple comparisons test was used. A *pvalue* < 0.05 was considered statistically significant." (Lines 255-257)

In the "**Abstract**" section:

"At a 15 mM HMF scale, this strategy achieved a FDCA yield of 100%, significantly higher than the 78.6% yield of the dual-enzyme cascade system established by HMFO and VDH1, and the 56.73% yield from the dual-strain catalysis constructed by two engineered *E. coli* strains that harbor either *hmfo* or *vdh*1 (Student's *t*-test, $p < 0.0001$)." (Line 29)

In the "**Results and Discussion**" section:

"Meanwhile, the growth curve of *E. coli* BL21/pET28a-sumo-*hmfo-vdh*1 during FDCA biosynthesis was monitored (Fig. S9)." (Line 391)

"As shown in Fig. 4, HMF was completely transformed to FDCA by neither the dual-

enzyme cascade system nor the dual-strain catalysis system.” (Line 448)

“Specifically, when compared separately with the dual-strain and dual-enzyme systems, this system showed 76.4% and 27.2% higher FDCA synthesis efficiency, respectively. Both comparisons were statistically significant (Student’s *t*-test, $p< 0.0001$).” (Lines 458-460)

“The results show that, in terms of FDCA synthesis, sequential regulation of dual catalytic modules is more efficient compared with the dual-strain catalysis and dual-enzyme cascade systems.” (Line 457)

In the **Figure** section:

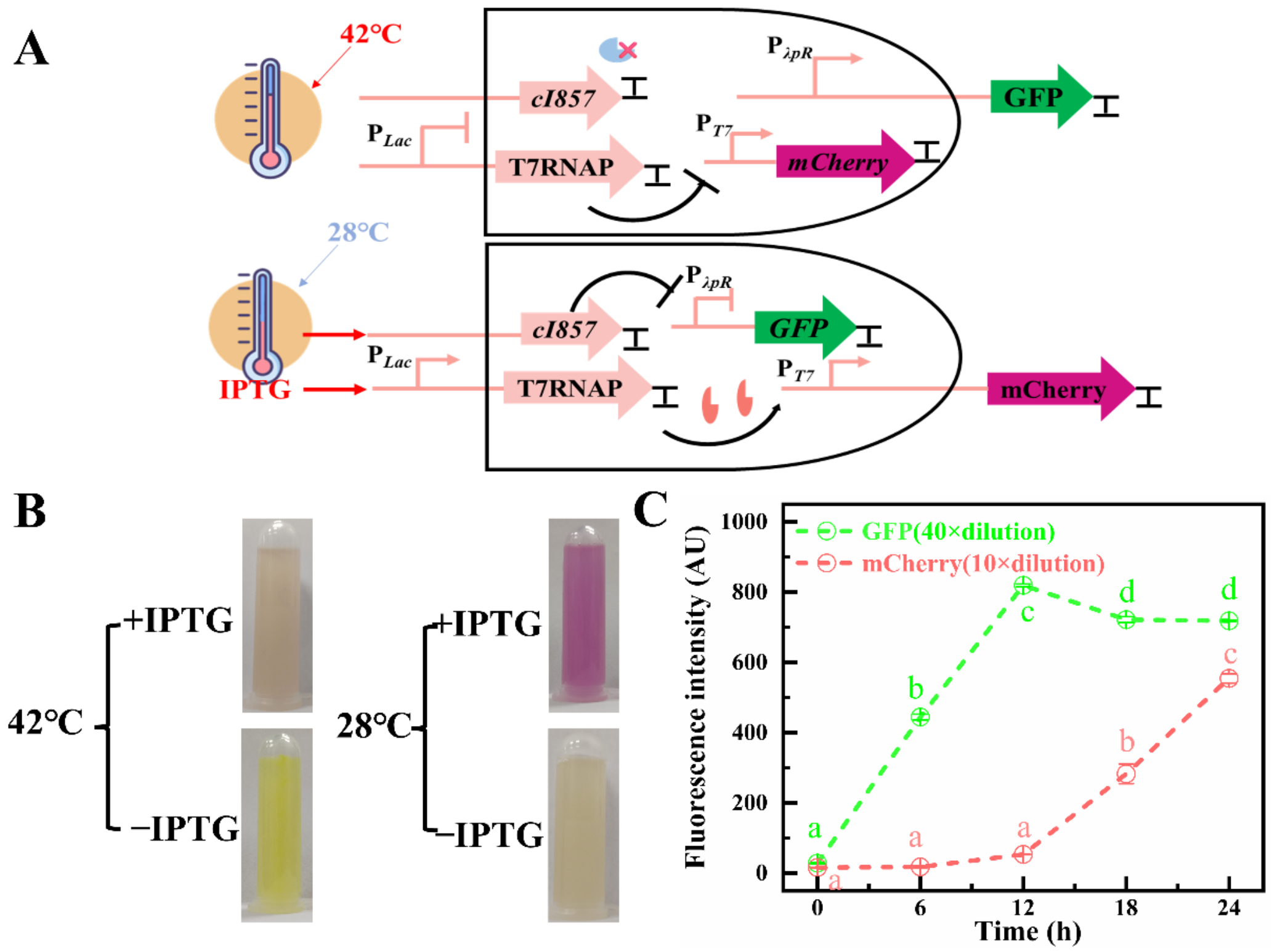

Fig. 1 Sequential regulation of dual catalytic modules in the present study. (A) Schematic diagram of sequential regulation of dual catalytic modules. (B) Sequential regulation of dual catalytic modules expressed GFP and mCherry at different temperatures. (C) The fluorescence intensity of mCherry and GFP, and the data are presented as the means ± SD of three independent experiments. Statistical comparisons were performed using one-way ANOVA followed by Tukey's multiple comparisons test. Different lowercase letters indicate significant differences among groups ($p < 0.05$). Groups sharing the same letter are not significantly different.

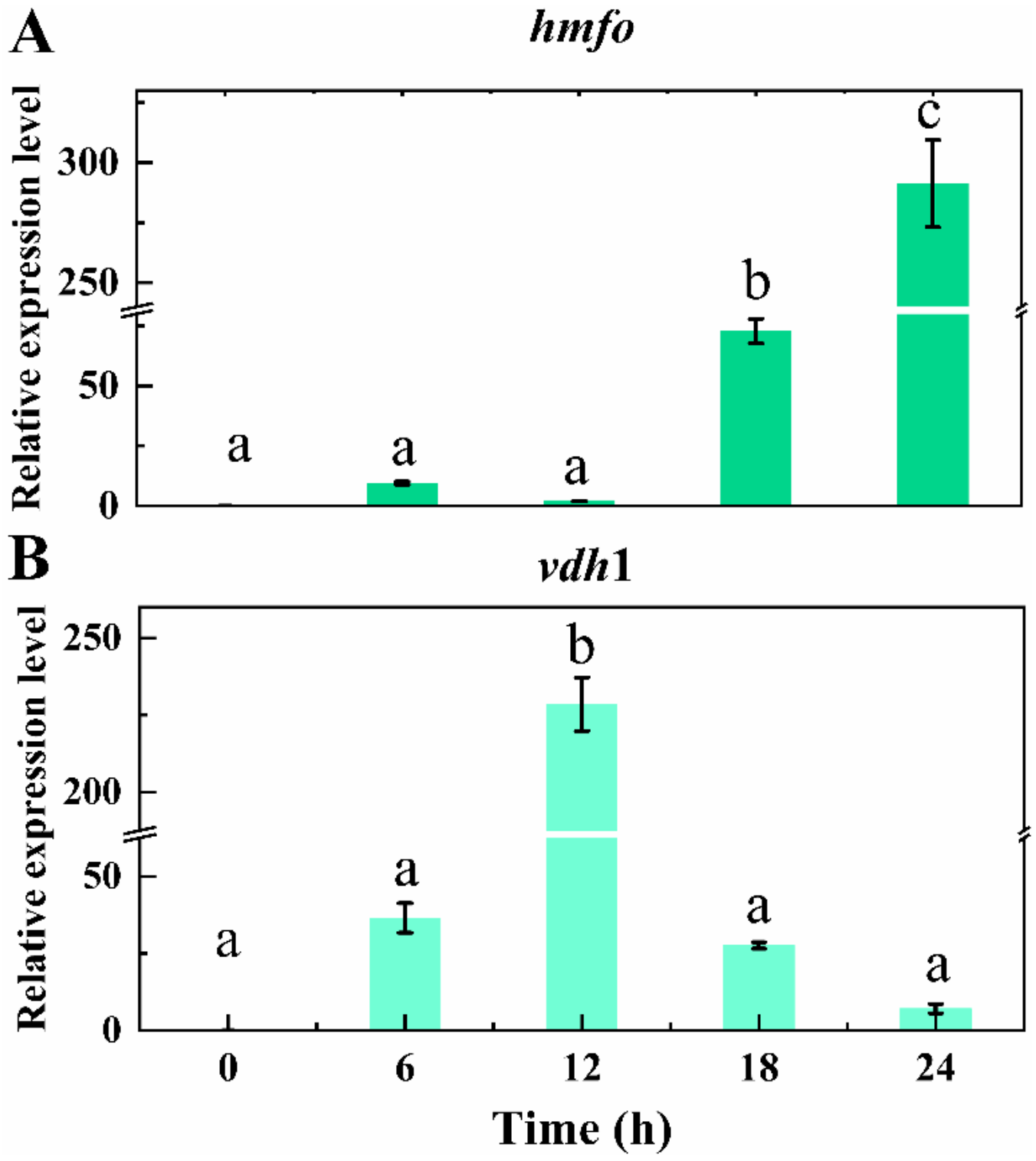

Fig. 2 The relative transcription levels of *hmfo* and *vdh*1 in sequential regulation of dual catalytic modules. 16S rRNA was used as the housekeeping gene for normalization. VDH1 was induced during 0–12 h by alternating incubation between 42 °C for 0.5 h and 37 °C for 3 h. HMFO was induced during 12–24 h by shifting to 18 °C. Data are presented as the means ± SD of three independent experiments. Statistical comparisons were performed using one-way ANOVA followed by Tukey's multiple comparisons test. Different lowercase letters indicate significant differences among groups ($p < 0.05$). Groups sharing the same letter are not significantly different.
