# Supplementary material for: Sequential regulation of dual catalytic modules for the directed biosynthesis of 2,5-furandicarboxylic acid in Escherichia coli
Source: Bioresour Bioprocess. 2026 Nov 7;13(1):163. doi: 10.1186/s40643-026-01159-5 (PMC13744578; doi:10.1186/s40643-026-01159-5)
Supplement: Supplementary file 2 [file 40643_2026_1159_MOESM1_ESM.docx]

Sequential regulation of dual catalytic modules for the directed biosynthesis of 2,5-furandicarboxylic acid in *Escherichia coli*

*Fan Yang ^a, b^, Yan Li ^a, b^, Xinrui Qi ^a, b^, Huanan Li ^a, b^, Wanli Cheng ^a, b^, Jiashu Liu ^a, b, †^, Zhengbing Jiang ^a, b, †^*

a School of Life Science, Hubei University, Wuhan,430062, P.R. China.

b Hubei Key Laboratory of Industrial Biotechnology, Hubei University, Wuhan,430062, P.R. China.

† Corresponding author

E-mail address: [jsliu@hubu.edu.cn](mailto:jsliu@hubu.edu.cn)

E-mail address: [zhbjiang@hubu.edu.cn](mailto:zhbjiang@hubu.edu.cn).

**Supporting Information**

**Supplementary Figures**

[Fig S1 The schematic diagram of the plasmid pET28a-*mcherry*-*gfp* construction	3](#_Toc243149442)

[Fig S2 System boundaries of the production of FDCA	4](#_Toc243149443)

[Fig S3 The effect of temperature on GFP and mCherry expression	4](#_Toc243149444)

[Fig S4 The growth curve of *E. coli* BL21/pET28a-sumo*-hmfo-vdh*1 under different culture temperatures	4](#_Toc243149445)

[Fig S5 SDS-PAGE analyzed the VDH1 and HMFO expression effect in BL21/pET28a-sumo-*hmfo-vdh*1.	4](#_Toc243149446)

[Fig S6 The growth curve of *E. coli* BL21/pET28a-sumo-*hmfo-vdh*1 under different HMF concentrations	5](#_Toc243149447)

[Fig S7 The conversion of HMF by VDH1 in the BL21/pET28a-sumo-*hmfo-vdh*1 under varying induction time..	5](#_Toc243149448)

[Fig S8 The concentration of FDCA by the BL21/pET28a-sumo-*hmfo-vdh*1 under varying temperatures.	6](#_Toc243149449)

[Fig S9 The growth curve of *E. coli* BL21/pET28a-sumo-*hmfo-vdh*1 during FDCA biosynthesis	6](#_Toc243149450)

[Fig S10 The concentration of FDCA by the BL21/pET28a-sumo-*hmfo-vdh*1 at varying HMF concentration.	7](#_Toc243149451)

[Fig S11 Thermal stability assessment of recombinant VDH1	7](#_Toc243149452)

[Fig S12 Process relative contributions (in %) per subsystem.	8](#_Toc243149453)

[Fig S13 Sensitivity coefficient of LCA to chemical inputs	8](#_Toc243149454)

**Supplementary Tables**

[Table S1 Synthesis of FDCA via various biocatalytic processes.	9](#_Toc243149313)

[Table S2 The plasmids and strains are used in this study	10](#_Toc243149314)

[Table S3 Primer sequence in this study	11](#_Toc243149315)

[Table S4 Life Cycle Inventory (LCI) for FDCA production (functional unit: Fermentation broth containing 100g FDCA) via sequential regulation of dual catalytic modules.	11](#_Toc243149316)

[Table S5 Life Cycle Inventory (LCI) for FDCA production (functional unit: Fermentation broth containing 100g FDCA) via the dual-strain catalysis systems.	12](#_Toc243149317)

[Table S6 Life Cycle Inventory (LCI) for FDCA production (functional unit: Fermentation broth containing 100g FDCA) via the dual-enzyme system.	12](#_Toc243149318)

[Table S7 Impact Categories for ReCiPe 2016 Midpoint Method	13](#_Toc243149319)

[Table S8 The uncertainty analysis data for LCA.	14](#_Toc243149320)

[Table S9 Sensitivity coefficient of LCA to batch duration	15](#_Toc243149321)

**Supplementary Figures**

### Fig S1 The schematic diagram of the plasmid pET28a-*mcherry*-*gfp* construction

### Fig S2 System boundaries of the production of FDCA (green arrows indicate inputs, blue arrows indicate outputs).

### Fig S3 The effect of temperature on GFP and mCherry expression

### Fig S4 The growth curve of *E. coli* BL21/pET28a-sumo*-hmfo-vdh*1 under different culture temperatures. *E. coli* was cultured at 37°C for the untreated control, while the experimental group underwent alternating incubation at 42 °C for 0.5 h and 37 °C for 3 h, for a total duration of 12 h.

Fig S5 SDS-PAGE analyzed the VDH1 and HMFO expression effect in BL21/pET28a-sumo-*hmfo-vdh*1. M: protein marker; lane 1: crude supernatant from induced BL21/pET28a cells; lane 2: crude VDH1 and HMFO supernatant; lane 3: crude HMFO supernatant; lane 4: crude VDH1 supernatant.

### Fig S6 The growth curve of *E. coli* BL21/pET28a-sumo-*hmfo-vdh*1 under different HMF concentrations

### Fig S7 The conversion of HMF by VDH1 in the BL21/pET28a-sumo-*hmfo-vdh*1 under varying induction time. The VDH1 expression was induced by a temperature alternation strategy, alternating between 42°C for 0.5 h and 37°C for 3 h as a cycle. (A) a cycle, (B) two cycles, (C) three cycles.

### Fig S8 The concentration of FDCA by the BL21/pET28a-sumo-*hmfo-vdh*1 under varying temperatures. The VDH1 was induced expression for 7 h at alternating between 42°C and 37°C, and HMFO was induced expression for 12 h at 18°C. The temperature for FDCA biosynthesis was analyzed at 25℃ and 35℃. (A)25℃. (B) 35℃.

### Fig S9 The growth curve of *E. coli* BL21/pET28a-sumo-*hmfo-vdh*1 during FDCA biosynthesis

### Fig S10 The concentration of FDCA by the BL21/pET28a-sumo-*hmfo-vdh*1 at varying HMF concentration. (A) 20 mM HMF, (B) 25 mM HMF

### Fig S11 Thermal stability assessment of recombinant VDH1

### Fig S12 Process relative contributions (in %) per subsystem. Step 1 (Strain cultivation), Step 2 (Induce expression), Step 3 (FDCA production). (A) sequential regulation of dual catalytic modules, (B) the dual-strain catalysis system, (C) the dual-enzyme cascade system.

### Fig S13 Sensitivity coefficient of LCA to chemical inputs. A. Sequential regulation of dual catalytic modules, B. The dual-strain catalysis systems, C. The dual-enzyme system

**Supplementary Tables**

### Table S1 Synthesis of FDCA via various biocatalytic processes.

| Biocatalyst strategies | Substrate | Biocatalyst | Reaction conditions | Titer (mM) | Yield (%) | Productivity mM h^-1^ | Time (h) | Ref. |
| --- | --- | --- | --- | --- | --- | --- | --- | --- |
| Sequential regulation of dual catalytic modules | 15 mM HMF | *E. coli_hmfo_vdh1* | TB medium, 30 °C, 200 rpm |  | 100 | 0.13 | 120 | This study |
|  | 20 mM HMF | *E. coli_hmfo_vdh1* | TB medium, 30 °C, 200 rpm |  | 88.95 | 0.15 | 120 |  |
|  | 25 mM HMF | *E. coli_hmfo_vdh1* | TB medium, 30 °C, 200 rpm |  | 75.52 | 0.26 | 72 |  |
| Microbial catalysis | 25 mM DFF | *E. coli_*AAOase | 0.1g/mL *E. coli_*AAOase, 10% DMSO, phosphate buffer (pH 8.0), 35 °C | ─ | 100 | 1.04 | 24 | [1] |
|  | 180 mM HMF | *E. coli*_HMFO,  *D. wulumuqiensis* R12 | 0.1 g/mL *E. coli*_HMFO, 0.2 g/mL of *D. wulumuqiensis* R12, Sodium phosphate buffer (100 mM, pH 7.4), 25 °C, and 850 rpm, 20 mg/mL CaCO_3_ | ─ | 99 | 2.48 | 72 | [2] |
|  | 80 mM HMF | *B. subtilis* J8M8_HMfH*_*ADH*_*MaAAO | *B. subtilis* J8M8_HMfH*_*ADH*_*MaAAO OD600=90, phosphate buffer (pH 7.0), 35 °C | ─ | 90.4 | 0.86 | 84 | [3] |
|  | 15g/L FFCA | *Gluconobacter oxydans* DSM 50049 | 200 mg/mL *Gluconobacter oxydans* DSM 50049, Sodium acetate buffer (100 mM, pH 5.0), DO 70%, 30 °C, 500 rpm | ─ | 90 | 11.34 | 8.5 | [4] |
| Microbial + enzymatic catalysis | 100 mM HMF | *Comamonas testosteroni* SC1588, Laccase, TEMPO | 70 mg/mL *Comamonas testosteroni* SC1588, 5.4 mg/mL Laccase, 20 mol% TEMPO, phosphate buffer (200 mM, pH 7.0), 30 °C, 160 rpm | ─ | 87 | 2.42 | 36 | [5] |
|  | 25 mM HMF | GO M3-5 CFE, HRP, catalase,  *E. coli_*VDH1*_*NOX | 0.5 U/mL GO M3-5 CFE, 5 mg/mL *E. coli_*VDH1*_*NOX, phosphate buffer (200 mM, pH 7), 0.05 mg/mL HRP, 0.1 mg/mL catalase, 0.5 mM CuCl_2_, 30 °C, 150 rpm | ─ | 90 | 0.63 | 36 | [6] |
| Enzymatic catalysis | 200 mM HMF | Laccase, TEMPO | 6 mg/mL Laccase, 20 mol% TEMPO, Citrate buffer (100 mM, pH 6.0), 30 °C, 150 rpm | ─ | 97 | 6.93 | 28 | [7] |
|  | 40 mM HMF | *Pseudomonas putida* KT2440_PedH cell lysates | *Pseudomonas putida* KT2440_PedH cell lysates, OD600=50, Tris-HCl (100 mM, pH 8), 30 μM PQQ, 30 μM PrCl3, 1 mM Wurster's blue, 30 °C | ─ | 96.4 | 0.80 | 48 | [8] |
|  | 50 mM HMF | *BP*1-Lip, *Cgl*AlcOx | 1.2 U/mL *BP*1-Lip, 24 U/mL *Cgl*AlcOx, phosphate buffer (50 mM, pH 7), 28 °C, 200 rpm | 41.03 | 82.06 | 0.16 | 264 | [9] |

### Table S2 The plasmids and strains are used in this study

| Plasmids and strains | Genotype—descriptions | Sources |
| --- | --- | --- |
| Plasmids |  |  |
| pET28a | P*_T7_* | lab stock |
| pBV220 | P*_R_*/P*_L_* | lab stock |
| pDawn-*mcherry* | Cloning the *mcherry* gene | lab stock |
| pET28a-*gfp* | Cloning the *gfp* gene | lab stock |
| pET28a-*mcherry* | Cloning the *mcherry* gene into *Bam*H I and *Not* I sites of pET28a | This study |
| pBV- *gfp* | Cloning *gfp* gene into *Bam*H I and *Eco*R I sites of pBV220 | This study |
| pET28a-*mcherry-gfp* | One-step cloning *cI857-P_R_/P_L_-gfp-terminator* segment in pET28a-*mcherry* | This study |
| pET28a-sumo-*hmfo* | *hmfo* gene was synthesized and constructed in pET28a-sumo | This study |
| pET28a-*vdh*1 | *vdh*1 gene was synthesized and constructed in pET28a | This study |
| pBV- *vdh1* | Cloning *vdh*1 gene into *Bam*H I and *Eco*R I sites of pBV220 | This study |
| pET28a-sumo-*hmfo-vdh*1 | One-step cloning *cI857-pR/pL-vdh*1*-terminator* segment in pET28a-sumo-*hmfo* | This study |
| Strains |  |  |
| BL21/ pBV- *gfp* | *E. coli* BL21(DE3), P*_R_*/P*_L_*: *gfp*. Kan | This study |
| BL21/pET28a-*mcherry* | *E. coli* BL21(DE3), P*_T7_*: *mcherry*. Kan | This study |
| BL21/pET28a-*mcherry-gfp* | *E. coli* BL21(DE3), P*_T7_*: *mcherry*. P*_R_*/P*_L_*: *gfp*. Kan | This study |
| BL21/pET28a-sumo-*hmfo* | *E. coli* BL21(DE3), P*_T7_*: *hmfo*. Kan | This study |
| BL21/pET28a-*vdh*1 | *E. coli* BL21(DE3), P*_T7_*: *vdh*1. Kan | This study |
| BL21/pET28a-sumo-*hmfo-vdh*1 | *E. coli* BL21(DE3), P*_T7_*: *hmfo*. P*_R_*/P*_L_*: *vdh*1. Kan | This study |

### Table S3 Primer sequence in this study

| Primer | Sequence | |
| --- | --- | --- |
| PET-mCherry-F | tggtgtgcggccgcttccttgtacagctc | |
| PET-mCherry-R | aaatgggtcgcggatccatggttagcaaagg | |
| PBV-GFP-F | ttaaggaggaattcatggtgagcaagggc | |
| PBV-GFP-R | cgacggatccccggttatttatacagtt | |
| T7-CI-F | ttcgcgggatcgatcagccaaacgtct | |
| PET-TER-R | gatggcctttttgcgatctcgatcctctac | |
| PBV-VDH-F | attaaggaggaattcatgatcgaacaga | |
| PBV-VDH-R | gacggatccccggttagaacgggta | |
| TER-PET-F | | gatggcctttttgcgatctcgatcctctac |
| CI-T7-R | agacgtttggctgatcgatcccgcgaa | |
| Q-VDH1-F | gtgcggcaacacggttatc | |
| Q-VDH1-R | gagcaacgatagcttccacaac | |
| Q-HMFO-F | cctgccgcgtgattatgatg | |
| Q-HMFO-R | tcagaccagtgacgggaatc | |
| 16S-F | catgccgcgtgtatgaagaa | |
| 16S-R | cgggtaacgtcaatgagcaaa | |

### Table S4 Life Cycle Inventory (LCI) for FDCA production (functional unit: Fermentation broth containing 100g FDCA) via sequential regulation of dual catalytic modules.

| Step | Input | Quantity | Unit |
| --- | --- | --- | --- |
| Step 1  Strain cultivation | Tryptone | 0.009337 | g |
|  | Yeast Extract | 0.0046685 | g |
|  | NaCl | 0.009337 | g |
|  | Kanamycin | 0.000047 | g |
|  | Water | 0.9337 | L |
|  | Electricity | 8.4 | kW·h |
| Step 2  Induce expression | Tryptone | 0.56022 | g |
|  | Yeast Extract | 1.12044 | g |
|  | Glycerol | 0.233425 | g |
|  | KH_2_PO_4_ | 0.1073755 | g |
|  | K_2_HPO_4_ | 0.765634 | g |
|  | Kanamycin | 0.002334 | g |
|  | Isopropyl-beta-D-thiogalactopyranoside | 0.00445 | g |
|  | Electricity | 445.6 | kW·h |
|  | Water | 46.685 | L |
| Step 3  FDCA production | HMF | 0.082328 | g |
|  | Electricity | 763.94 | kW·h |

### Table S5 Life Cycle Inventory (LCI) for FDCA production (functional unit: Fermentation broth containing 100g FDCA) via the dual-strain catalysis systems.

| Step | Input | Quantity | Unit |
| --- | --- | --- | --- |
| Step 1  Strain cultivation | Tryptone | 0.03012 | g |
|  | Yeast Extract | 0.01506 | g |
|  | NaCl | 0.03012 | g |
|  | Kanamycin | 0.0001506 | g |
|  | Water | 3.012 | L |
|  | Electricity | 8.4 | kW·h |
| Step 2  Induce expression | Tryptone | 1.8072 | g |
|  | Yeast Extract | 3.6144 | g |
|  | Glycerol | 0.753 | g |
|  | KH_2_PO_4_ | 0.34638 | g |
|  | K_2_HPO_4_ | 2.46984 | g |
|  | Kanamycin | 0.00753 | g |
|  | Isopropyl-beta-D-thiogalactopyranoside | 0.010766 | g |
|  | Electricity | 821.45 | kW·h |
|  | Water | 150.6 | L |
| Step 3  FDCA production | HMF | 0.082328 | g |
|  | Na_2_HPO_4_ | 0.3084288 | g |
|  | NaH_2_PO_4_·2H_2_0 | 0.24849 | g |
|  | Water | 75.3 | L |
|  | Electricity | 3285.82 | kW·h |

### Table S6 Life Cycle Inventory (LCI) for FDCA production (functional unit: Fermentation broth containing 100g FDCA) via the dual-enzyme system.

| Step | Input | Quantity | Unit |
| --- | --- | --- | --- |
| Step1  Strain cultivation | Tryptone | 0.0435 | kg |
|  | Yeast Extract | 0.02175 | kg |
|  | NaCl | 0.0435 | kg |
|  | Kanamycin | 0.0002175 | kg |
|  | water | 4.35 | L |
|  | Electricity | 8.4 | kW·h |
| Step2  Induce expression  Protein purification | Tryptone | 2.17392 | kg |
|  | Yeast Extract | 1.08696 | kg |
|  | NaCl | 3.69845 | kg |
|  | Kanamycin | 0.0108696 | kg |
|  | Isopropyl-beta-D-thiogalactopyranoside | 0.01295 | kg |
|  | Na_2_HPO4 | 5.17693 | kg |
|  | NaH_2_PO_4_·2H_2_0 | 3.637418 | kg |
|  | Imidazole | 7.16322 | kg |
|  | lysozyme | 0.1304352 | kg |
|  | Electricity | 3481.8972 | kW·h |
|  | water | 1413.048 | L |
| Step3  FDCA production | HMF | 0.082328 | kg |
|  | Na_2_HPO_4_ | 0.3084288 | kg |
|  | NaH_2_PO_4_·2H_2_0 | 0.235315 | kg |
|  | water | 54.348 | L |
|  | Electricity | 1304.352 | kW·h |

### Table S7 Impact Categories for ReCiPe 2016 Midpoint Method

| Impact Category | Abbreviations | Units |
| --- | --- | --- |
| Terrestrial acidification potential | TAP | kg SO2-eq |
| Global warming potential | GWP 100 | kg CO2-eq |
| Freshwater ecotoxicity potential | FETP | kg 1,4-DCB-eq |
| Marine ecotoxicity potential | METP | kg 1,4-DCB-eq |
| Terrestrial ecotoxicity potential | TETP | kg 1,4-DCB-eq |
| Fossil fuel potential | FFP | kg oil-eq |
| Freshwater eutrophication potential | FEP | kg P-eq |
| Marine eutrophication potential | MEP | kg N-eq |
| Human toxicity potential: carcinogenic | HTPc | kg 1,4-DCB-eq |
| Human toxicity potential: non-carcinogenic | HTPnc | kg 1,4-DCB-eq |
| Ionising radiation potential | IRP | kBq Co-60-eq |
| Agricultural land occupation | LOP | m2*a crop-eq |
| Surplus ore potential | SOP | kg Cu-eq |
| Ozone depletion potential | ODP | kg CFC-11-eq |
| Particulate matter formation potential | PMFP | kg PM2.5-eq |
| Photochemical oxidant formation potential: humans | HOFP | kg NOx-eq |
| Photochemical oxidant formation potential: ecosystems | EOFP | kg NOx-eq |
| Water consumption potential | WCP | m^3^ |

Table S8 The uncertainty analysis data for LCA.

|  | Freshwater ecotoxicity potential (FETP) median [90% CI] kg 1,4-DCB-eq | Marine ecotoxicity potential (METP) median [90% CI] kg 1,4-DCB-eq | Terrestrial ecotoxicity potential (TETP) median [90% CI] kg 1,4-DCB-eq | Global warming potential median [90% CI]  kg CO₂-eq | Fossil fuel potential median [90% CI]  kg oil-eq |
| --- | --- | --- | --- | --- | --- |
| Sequential regulation of dual catalytic modules | 42.90  [38.89, 46.49] | 56.20  [51.02, 60.98] | 2562.71  [2328.92, 2760.65] | 909.07  [825.25, 988.87] | 183.52  [166.59, 199.61] |
| Dual-strain catalysis system | 144.99  [129.18, 158.65] | 189.62  [169.47, 208.11] | 8666.68  [7727.00, 9401.57] | 3065.03  [2739.03, 3373.70] | 618.84  [552.92, 681.01] |
| Dual-enzyme cascade systems | 230.25  [208.58, 251.46] | 302.88  [274.48, 330.70] | 14161.20  [12930.11, 15366.52] | 4886.73  [4420.14, 5343.55] | 994.90  [900.73, 1087.09] |

Table S9 Sensitivity coefficient of LCA to batch duration

|  | GWP | FETP | METP | TETP | FFP |
| --- | --- | --- | --- | --- | --- |
| Sequential regulation of dual catalytic modules | 0.61 | 0.60 | 0.60 | 0.57 | 0.61 |
| The dual-strain catalysis systems | 0.78 | 0.77 | 0.77 | 0.73 | 0.78 |
| The dual-enzyme system | 0.27 | 0.26 | 0.26 | 0.24 | 0.26 |

**Reference**

1.	Yang, Q.; Wu, C.; Zhang, T.; He, Y. C.; Ma, C., Efficient bio-oxidation of biomass-derived furan-2,5-dicarbaldehyde to 5-formyl-2-furoic acid and 2,5-furandicarboxylic acid via whole-cell biocatalysis. *Bioresour Technol* **2025,** *421*, 132201. <https://doi.org/10.1016/j.biortech.2025.132201>.

2.	Zhang, Z.-G.; Yi, Y.; Lin, J.-C.; Chen, D.; Ji, X.-J., One-pot two-step biocatalytic upgrading of 5-hydroxymethylfurfural to 2, 5-furandicarboxylic acid (FDCA) through a sequential oxidation process. *Sustainable Chemistry and Pharmacy* **2024,** *39*, 101616. <https://doi.org/https://doi.org/10.1016/j.scp.2024.101616>.

3.	Jiang, X.; Chen, C.; Jiao, M.; He, J.; Han, X.; Wang, T.; Huang, D.; Jiang, Y.; Liu, H.; Yuan, H. Whole-Cell Biocatalytic Production of 2,5-Furandicarboxylic Acid from 5-Hydroxymethylfurfural by *Bacillus subtilis* J8M8 *Microorganisms* 2026.14(2):425. https://doi.org/10.3390/microorganisms14020425

4.	Sayed, M.; Ismail, M.; Sivasubramanian, A.; Kawano, R.; Li, C.; Glaser, S. J.; Hatti-Kaul, R., *Gluconobacter oxydans* DSM 50049 – an efficient biocatalyst for oxidation of 5-formyl-2-furancarboxylic acid (FFCA) to 2,5-furandicarboxylic acid (FDCA). *Microbial Cell Factories* **2025,** *24* (1), 68. <https://doi.org/10.1186/s12934-025-02689-x>.

5.	Yang, Z.-Y.; Wen, M.; Zong, M.-H.; Li, N., Synergistic chemo/biocatalytic synthesis of 2,5-furandicarboxylic acid from 5-hydroxymethylfurfural. *Catalysis Communications* **2020,** *139*, 105979. <https://doi.org/https://doi.org/10.1016/j.catcom.2020.105979>.

6.	Zhu, F.-F.; Wang, J.-P.; Zong, M.-H.; Zheng, Z.-J.; Li, N., Concurrent Biocatalytic Oxidation of 5-Hydroxymethylfurfural into 2,5-Furandicarboxylic Acid by Merging Galactose Oxidase with Whole Cells. *Processes* **2023,** *11* (8), 2261. <https://doi.org/10.3390/pr11082261>.

7.	Cheng, A.-D.; Zong, M.-H.; Lu, G.-H.; Li, N., Solvent-Promoted Oxidation of Aromatic Alcohols/Aldehydes to Carboxylic Acids by a Laccase-TEMPO System: Efficient Access to 2,5-Furandicarboxylic Acid and 5-Methyl-2-Pyrazinecarboxylic Acid. *Advanced Sustainable Systems* **2021,** *5* (6), 2000297. <https://doi.org/https://doi.org/10.1002/adsu.202000297>.

8.	Liu, K.; Jiang, L.; Wang, L.; Zhang, Q.; Yang, L.; Wu, J.; Yu, H., Sequential single-enzyme oxidation of 5-hydroxymethylfurfural to 2,5-furandicarboxylic acid by an engineered lanthanide-dependent alcohol dehydrogenase. *Green Chemistry* **2025,** *27* (18), 5073-5090. <https://doi.org/10.1039/D5GC00157A>.

9.	Li, Y.; Yang, F.; Tan, X.; Li, Y.; Gong, C.; Li, H.; Cheng, W.; Xiao, W.; Liu, J.; Jiang, Z., Lipase and alcohol oxidase cascade catalytic system for the efficient conversion of 5-hydroxymethylfurfural to 2,5-furandicarboxylic acid. *International Journal of Biological Macromolecules* **2025,** *334*, 149081. <https://doi.org/https://doi.org/10.1016/j.ijbiomac.2025.149081>.
